# Activation chemistry drives the emergence of functionalised protocells†

**DOI:** 10.1039/d0sc04506c

**Published:** 2020-10-02

**Authors:** Claudia Bonfio, David A. Russell, Nicholas J. Green, Angelica Mariani, John D. Sutherland

**Affiliations:** Medical Research Council Laboratory of Molecular Biology Cambridge Biomedical Campus, Francis Crick Avenue Cambridge CB2 0QH UK bonfio@mrc-lmb.cam.ac.uk

## Abstract

The complexity of the simplest conceivable cell suggests that the chemistry of prebiotic mixtures needs to be explored to understand the intricate network of prebiotic reactions that led to the emergence of life. Early cells probably relied upon compatible and interconnected chemistries to link RNA, peptides and membranes. Here we show that several types of vesicles, composed of prebiotically plausible mixtures of amphiphiles, spontaneously form and sustain the methyl isocyanide-mediated activation of amino acids, peptides and nucleotides. Activation chemistry also drives the advantageous conversion of reactive monoacylglycerol phosphates into inert cyclophospholipids, thus supporting their potential role as major constituents of protocells. Moreover, activation of prebiotic building blocks within fatty acid-based vesicles yields lipidated species capable of localising to and functionalising primitive membranes. Our findings describe a potentially prebiotic scenario in which the components of primitive cells undergo activation and provide new species that might have enabled an increase in the functionality of protocells.

## Abstract 2

The complexity of the simplest conceivable cell suggests that the chemistry of prebiotic mixtures needs to be explored to understand the intricate network of prebiotic reactions that led to the emergence of life.

## Graphical abstract

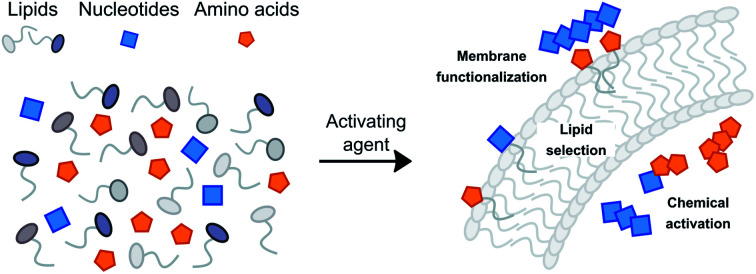

## Introduction

Cells are maintained by a diverse ensemble of interconnected molecular networks.^1^ Analogously, primitive cells must also have relied on a set of mutually compatible chemical processes for the synthesis of building blocks, the elongation of RNA and peptides and the assembly of compartments. Prebiotic systems chemistry aims to capture the complexity that emerges from chemical mixtures, thereby providing an experimental framework to better understand how life emerged and evolved on our planet.^2–5^ Recent systems chemistry reports have shown how prebiotic scenarios could have sustained the synthesis of nucleotides, amino acids, lipids and metal-based cofactors.^6–9^ These prebiotic components have been studied in various combinations in investigations of contemporary biological processes, such as nucleic acid–protein interactions and protein-mediated transport across membranes.^9–13^ However, the components of these different subsystems are most often studied in isolation, or, when they are mixed, their reactivities are programmed to avoid cross-reactions.^13^ To provide a more complete description of how primitive cells (protocells) could have emerged, experiments need to be performed that take into account the diverse reservoir of coexisting reactive species present on early Earth and do not preclude the potential formation of products of cross-reactivity, which may actually have been fundamental to the emergence of life.

Recent reports from our lab have shown how a systems chemistry approach combining atmospheric, inorganic and organic chemistries led to the identification of methyl isocyanide (MeNC) as a potentially prebiotic activating reagent. MeNC drives the simultaneous activation of nucleotides and peptides in aqueous solution.^14,15^ However, isonitrile-mediated activation chemistries on nucleotides and peptides in the presence of vesicles remain to be explored. We therefore sought to answer the following interrelated questions.

(i) Which vesicular systems can sustain the activation of prebiotic building blocks?

(ii) To what extent are vesicles altered by the activation chemistry occurring in and around them?

(iii) Do prebiotic lipids bearing a phosphate or carboxylate headgroup interfere with the activation of nucleotides and peptides?

(iv) Are novel and functional amphiphilic species formed in the process?

Here we demonstrate that vesicles made of primitive fatty acids or phospholipids are compatible with the conditions required for the simultaneous activation of prebiotic building blocks. These vesicles exhibit exceptional stability under the conditions employed and their constituent monomers only marginally affect the efficiency of activation reactions on nucleotides and peptides. Furthermore, MeNC was shown to drive the selective and advantageous conversion of monoacylglycerol phosphates into their cyclic phosphate derivatives, which are inert towards the activation chemistries tested. Our results highlight the compatibility of different protocellular constituents under activation conditions and show that prebiotically plausible vesicles could have coexisted with nucleotides and peptides, while allowing activation chemistries to produce RNA, peptides and peptidyl-RNA derivatives. Finally, activation of fatty acids in the presence of nucleotides or amino acids leads to novel lipid–nucleotide and lipid–amino acid adducts that functionalise the vesicle membranes, localise on their surface and induce their growth. The products derived from the condensation of nucleotides, amino acids and the constituent monomers of lipid membranes may have played a significant role in the selection and early evolution of functional protocells.

## Results and discussion

### Stable fatty acid-based vesicles form under the acidic conditions required for activation chemistry

**Fig. 1:**
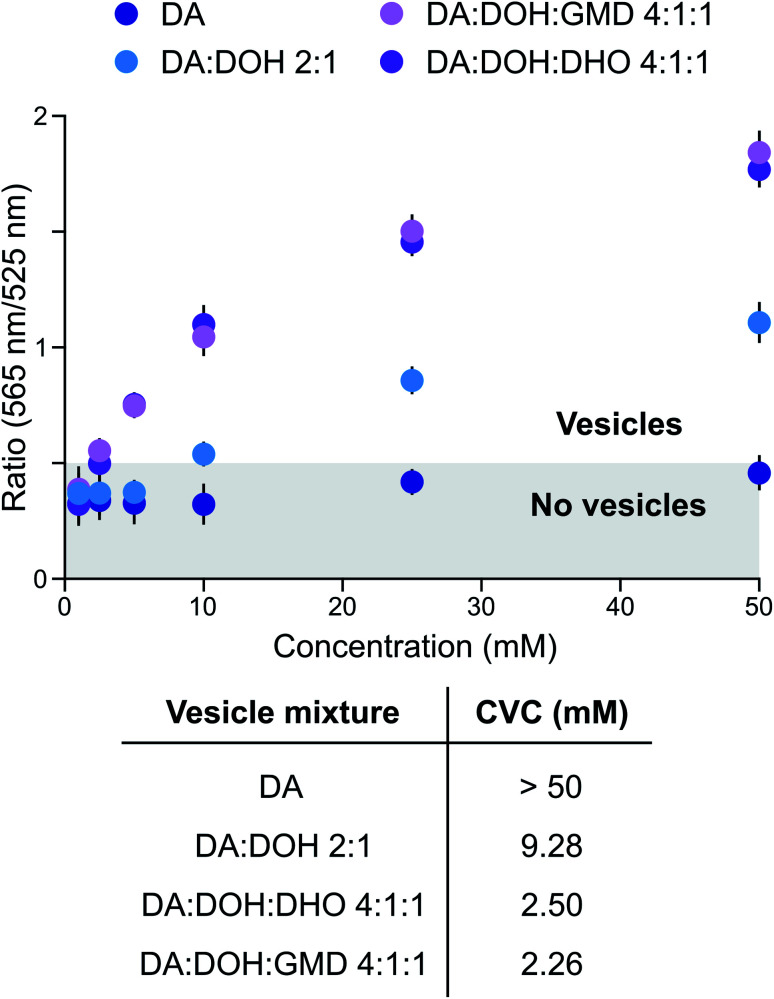
Evaluation of the stability of fatty acid-based membranes under activation conditions. CVC determination of DA-based vesicles in DCI buffer, pH 6. Data are representative of three independent experiments and values are expressed in mean ± SEM.

Prebiotically plausible fatty acids only form vesicles in a narrow window of neutral-to-alkaline pH values.^16^ For example, decanoic acid (DA, apparent p*K*_a_ ∼ 7 in membranes) forms vesicles around neutral pH, while in combination with other amphiphiles, such as decanol (DOH), vesicles also form at alkaline pH (up to ∼9).^17^ The formation and stability of DA-based vesicles under acidic conditions, however, have not been comprehensively explored. Since activation chemistry with MeNC can take place between pH 4 and 8,^14,15^ we investigated the self-assembly of prebiotic fatty acids in this pH range and evaluated the stability of vesicles formed thereof. Using 4,5-dicyanoimidazole (DCI), a co-product in the synthesis of adenine^18,19^ and a catalyst in our activation schemes,^15^ as a buffer between pH 4 and 6, DA did not form vesicles. This was confirmed by UV-Vis spectrophotometry performed in the presence of merocyanine 540, a lipophilic probe sensitive to lipid packing^20^ (Fig. 1). As we have recently shown that fatty aldehydes, acids and alcohols, derive from the homoaldolization of acetaldehyde,^6,21^ we next evaluated the ability of mixtures of C_10_-amphiphiles to self-assemble under acidic conditions. A mixture of DA, DOH, and decanal (DHO) in a 4 : 1 : 1 ratio led to the formation of vesicles at pH 6 in DCI buffer (Fig. 1). A small population of vesicles could still be observed at pH 5.5, alongside oil droplets and aggregates. To the best of our knowledge, this is the first example of a prebiotically plausible DA-based vesicle system that is stable down to approximately pH 5.5. This result confirms^22^ that vesicles could have formed from a complex mixture of amphiphiles likely present on early Earth. Moreover, these vesicles have a lower critical vesicle concentration (CVC) than those composed of homogenous fatty acids,^17^ and are stable over a broader pH range. Similar results were observed with different buffers and for precedented mixtures of DA with DOH (DA : DOH 2 : 1 ratio, pH 6) or with glycerol monodecanoate (GMD) (DA : DOH : GMD 4 : 1 : 1 ratio, pH 6) (Fig. 1). In particular, while the CVC of DA : DOH : DHO (4 : 1 : 1 ratio) vesicles in 2-(*N*-morpholino)ethanesulfonic acid (MES) buffer is around 50 mM at pH 6, we observed a 10 and 20-fold decrease of the CVC when imidazole or DCI were employed as buffers, respectively (Fig. S1†). Thus, DA-based vesicles spontaneously form under the conditions required for MeNC-mediated activation chemistry to take place and are stabilised by DCI and imidazole – molecules which may have served as catalysts in many prebiotic processes.

### Prebiotic building blocks are efficiently activated in the presence of fatty acid-based vesicles

**Fig. 2:**
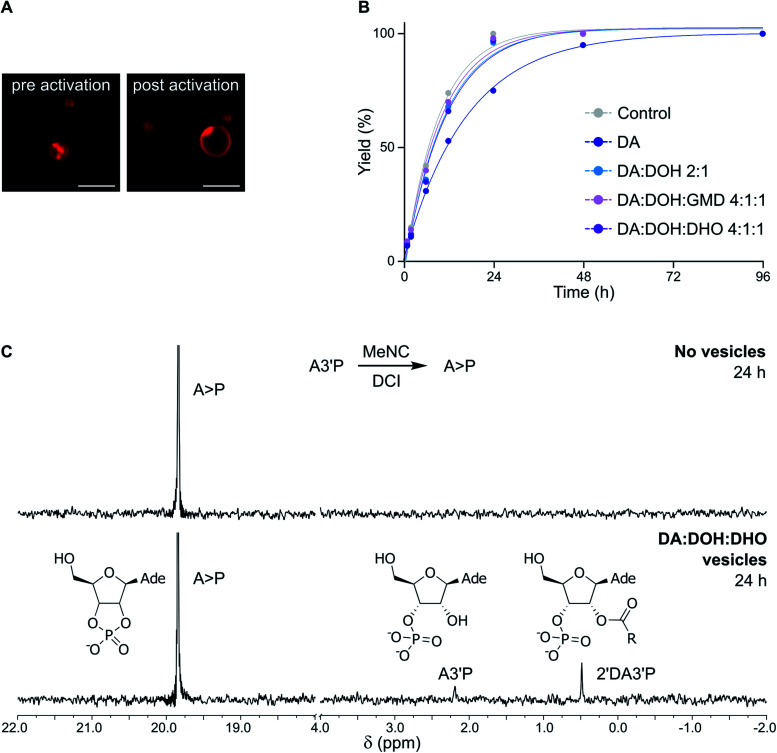
Evaluation of the compatibility of fatty acid-based vesicles and activation chemistry. (A) Confocal micrographs showing DA : DOH : DHO (4 : 1 : 1 ratio) vesicles in DCI buffer, pH 6, upon exposure to MeNC (samples were imaged before and after 96 h following the addition of the activating agent). (B) Plot showing the total yield (%) of products deriving from the activation of 10 mM A3′P (as determined by ^31^P-NMR spectroscopy) in the presence of 25 mM DA (dark blue) or vesicles made of DA : DOH (2 : 1 ratio) (light blue), DA : DOH : GMD (4 : 1 : 1 ratio) (pink) or DA : DOH : DHO (4 : 1 : 1 ratio) (violet), 25 mM total lipid concentration, in 100 mM DCI buffer, pH 6. (C) ^31^P-NMR spectra showing the activation of A3′P by MeNC after 24 h in the absence (top) or presence (bottom) of DA : DOH : DHO (4 : 1 : 1 ratio) vesicles in DCI buffer, pH 6.

To evaluate whether the constituents of fatty acid-based vesicles interfere with nucleotide phosphate activation chemistry, we studied the MeNC-mediated cyclization of adenosine 3′-monophosphate (A3′P) to adenosine 2′,3′-cyclic phosphate (A > P), in the presence of DA-based vesicles at pH 6. We have recently shown that nucleoside phosphates can be activated by MeNC in imidazole or DCI buffer in combination with acetaldehyde under near-to-neutral conditions (Passerini conditions)^14^ or under acidic conditions.^15^ In the presence of acetaldehyde, the nitrilium species formed by attack of MeNC can also be trapped by a carboxylate and the resulting intermediate can rearrange to form an ester (Fig. S2†). In the absence of aldehydes, protonation of MeNC can generate a nitrilium species, but the transient imidoyl carboxylate derived from this species cannot rearrange to an ester and so can instead be trapped by a nucleophile (other than water) or else is hydrolysed (Fig. S2†). While the combined presence of acetaldehyde and MeNC would thus be expected to induce the degradation of DA-based vesicles, sequestering fatty acids as esters^23^ incapable of self-assembly, fatty acid bilayers should not be affected by acid-catalysed activation (MeNC) in the absence of aldehydes. We tested this hypothesis by adding MeNC and acetaldehyde to a solution of A3′P in the presence of DA : DOH : GMD (4 : 1 : 1 ratio, pH 6) vesicles in imidazole buffer at pH 6.5, and, as expected, the vesicles were degraded (Fig. S3†). We then determined whether DHO, one of the components of the fatty acid-based vesicles employed in our studies, could react with MeNC in place of acetaldehyde. When 400 mM DHO was added to a solution of A3′P in imidazole buffer at pH 6.5, 14% of A > P was detected after 2 h by ^31^P-NMR spectroscopy. By comparison, A3′P was quantitatively converted into A > P after 0.5 h using 400 mM acetaldehyde (Fig. S4†). When the reaction was repeated using 4 mM DHO, a concentration equivalent to that available in 25 mM DA : DOH : DHO (4 : 1 : 1 ratio) vesicles at pH 6.5, 10% of A > P was detected after 48 h. This result was comparable to that obtained in the control reaction performed without either aldehyde, and therefore reflects the extent of activation brought about by the nitrilium species formed on protonation of MeNC in imidazole buffer (Fig. S5†). Taken together, these results indicate that the DHO present in the lipid bilayer contributes minimally, if at all, to nucleotide phosphate activation. Indeed, DA : DOH : DHO (4 : 1 : 1 ratio) vesicles exposed to MeNC were stable for at least 96 h in DCI buffer by confocal microscopy (Fig. 2A).

In the presence of DA : DOH : DHO (4 : 1 : 1 ratio) vesicles, the acid-catalysed activation (MeNC) of A3′P at pH 6 yielded less than 25% A > P after 24 h in the absence of DCI, as determined by ^31^P-NMR spectroscopy (Fig. S6†). This result is in agreement with that observed previously when the reaction was performed in the absence of vesicles.^15^ No products deriving from the activation of decanoic acid could be detected. However, when the reaction was repeated in DCI buffer, more than 90% of A > P was detected after 24 h (Fig. 2B, C and S7†), similar in efficiency to the reaction performed without vesicles. When the concentration of A3′P was reduced to 1 mM and 0.1 mM, to better mimic the oligonucleotide concentrations commonly employed in non-enzymatic RNA polymerisation reactions, complete conversion of A3′P into A > P was observed after 24 and 12 h, respectively (Fig. S8†). Additionally, formation of A > P was slower when lower concentrations of MeNC were used (Fig. S9†). These results are consistent with the expected more efficient cyclisation of the nucleotide at higher MeNC to nucleotide ratios. Moreover, increasing the pH of the solution also resulted in the slower formation of A > P (Fig. S10†), supporting the proposed acid-catalysed mechanism.^15^ Overall, this study demonstrates that DA-based vesicles are compatible with potentially prebiotic activating pathways.

### Activation chemistry in vesicula drives the formation of lipidated species

Once encapsulated within primitive cells, RNA, peptides, and lipids could have been combined upon activation to produce new species required for the emergence of more advanced systems. For example, the addition of lipids to nucleotides or peptides could have aided the localisation of (proto)biomolecules to the membrane, reminiscent of contemporary biochemical modifications such as myristoylation.^24^ We thus investigated whether fatty acids might be activated and subsequently trapped by nucleotides or amino acids to yield membrane-anchoring derivatives, without affecting the stability of primitive membranes.

Oligopeptides form when mixtures of amino acids and *N*-acylated amino acids are exposed to activation conditions.^15^ The formation of peptides results from attack of the amino group of the amino acid on the activated carboxylate group of the *N*-acylated amino acid and thence that of the growing peptide chain. As fatty acid carboxylates can be similarly activated,^15^ we wondered if the activated DA, originating from the DA : DOH : DHO (4 : 1 : 1 ratio) vesicles, could interfere with amino acid oligomerisation. Arginine was chosen as a model amino acid for its relevance in the prebiotic context due to its ability to electrostatically interact with negatively charged biological molecules, including RNA.^25,26^ Activation (MeNC) of arginine (Arg) in the presence of DA : DOH : DHO (4 : 1 : 1 ratio) vesicles, which was followed by ^1^H-NMR spectroscopy and mass spectrometry (Fig. S11 and S12†), led to the formation of *N*-methyl formamidinylated Arg (*N*-MFArg) oligomers. *N*-Decanoyl Arg oligomers (up to 3-mers) deriving from the activation of DA were also observed. The formation of *N*-decanoyl arginine (*N*-DecArg) was confirmed by mass spectrometry and further established by comparison of its NMR spectroscopic data with those of an authentic standard^27^ (Fig. S11 and S13†).

Activation (MeNC) of A3′P in DCI buffer at pH 6 in the presence of DA : DOH : DHO (4 : 1 : 1 ratio) vesicles gave, in addition to A > P, a minor product, assigned as 2′-decanoyl A3′P (2′DA3′P) (Fig. 2C, S14 and S15†). The identity of this species was confirmed by synthesis of an authentic standard and comparison of its NMR spectroscopic data (Fig. S16 and S18†). This lipidated nucleotide, which results from the activation of decanoic acid and its trapping by A3′P, was first observed after 6 h and continued to accumulate for up to 24 h, reaching a maximum yield of around 10% (Fig. S19 and S22†). The intramolecular cyclisation of the activated nucleotide is favored over the intermolecular reaction between the unactivated nucleotide and the activated DA, and, accordingly, 2′DA3′P could only be observed when a sufficiently high concentration of A3′P (>1 mM) was employed (Fig. S8†).

**Fig. 3:**
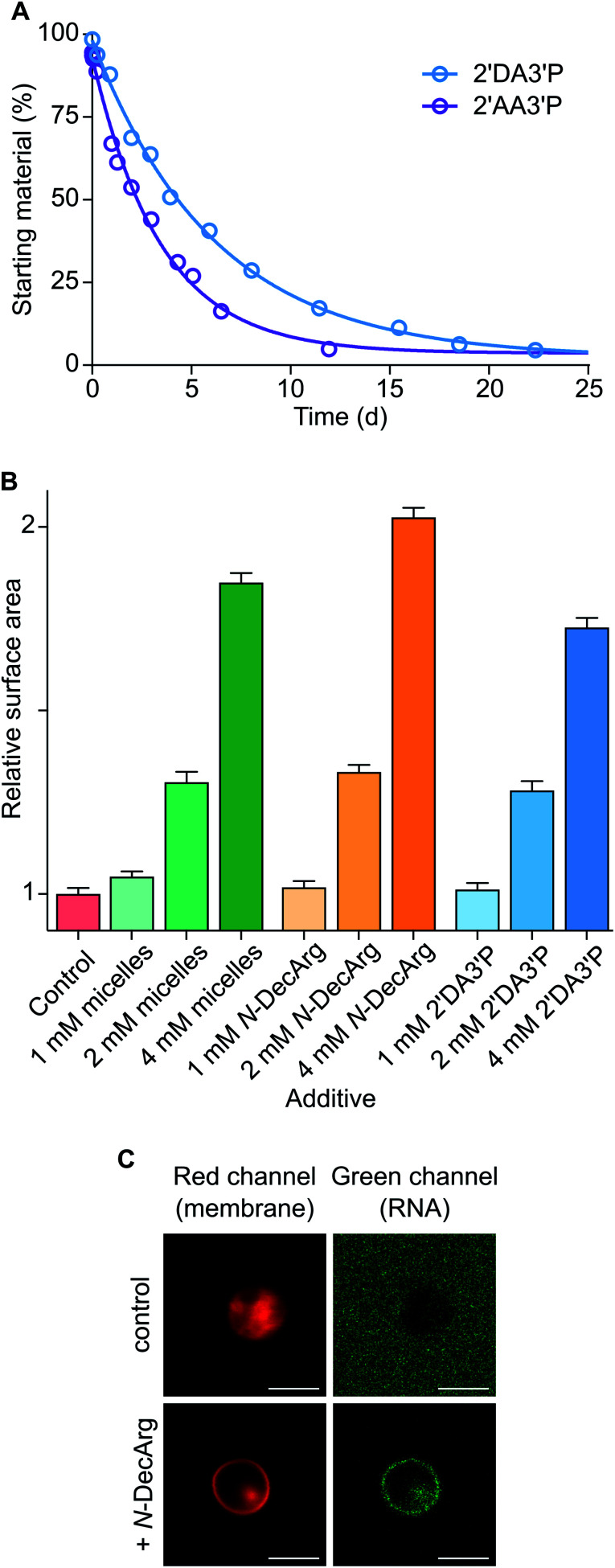
Characterisation and exploitation of lipidated nucleotides and amino acids. (A) Plot showing the hydrolysis profiles of 2′DA3′P and 2′AA3′P in DCI buffer, pH 6. (B) OA membrane growth upon addition of oleate micelles, *N*-DecArg, or 2′DA3′P. Control addition of DCI buffer is reported. (C) Confocal micrographs of a FAM-labeled 7-mer RNA oligonucleotide (green) localised to giant vesicles composed of mono-decanoylglycerol-1,2-cyclic phosphate and DOH (2 : 1 ratio, pH 8.5). Nile red dye (red) was used to label vesicle membranes. Top row shows results obtained in the absence of *N*-DecArg (control), bottom row shows results obtained in the presence of *N*-DecArg (25 mol%). Images are contrast adjusted. Scale bars represent 5 μm. Data are representative of three independent experiments and values are expressed in mean ± SEM.

To determine the persistence of 2′DA3′P, its hydrolysis was followed by HPLC and its half-life was calculated to be ∼5 days (Fig. 3A and S23†). Interestingly, acylated derivatives of A3′P have previously been shown to form with hydrophilic carboxylic acids or *N*-protected amino acids, however their half-lives were found to be markedly shorter (∼2 days and 2.5 h for the adducts formed with acetic acid (2′AA3′P, Fig. S24–26†) and *N*-acetylglycine (AcGly),^15^ respectively). The differences in the rates of hydrolysis of these species suggest that 2′DA3′P might form micellar systems or embed into the membranes of existing vesicles, in which it is partially protected from hydrolysis.

### Lipidated nucleotides and amino acids mediate the emergence of functionalised protocells

A prebiotic pathway that promotes the recruitment of biological building blocks to membranes could potentially facilitate intermolecular interactions and catalytic processes that would otherwise be hindered by excessive dilution. Recent reports show that pre-synthesised amphiphilic molecules and cationic *N*-carboxyanhydride-derived oleate-peptides are capable of anchoring to lipid bilayers.^25,28^ We therefore determined whether the 2′DA3′P and *N*-DecArg produced by activation (MeNC) are capable of inducing membrane growth by embedding into the lipid bilayer. The localisation of 2′DA3′P and *N*-DecArg to fatty acid and phospholipid membranes and the relative increase in membrane surface area were monitored using a Förster Resonance Energy Transfer (FRET) assay by labeling the lipid bilayers with a FRET donor–acceptor fluorescent dye pair (*N*-(7-nitro-2,1,3-benzoxadiazol-4-yl) (NBD) and lissamine rhodamine), as previously described^25^ (standard curves: Fig. S27†). An increase in the membrane surface area results from the insertion of amphiphiles into the bilayer and leads to a decrease in the FRET efficiency. In experiments performed with oleic acid (OA) vesicles, we observed a decrease in the FRET signal, which correlated with an increase in the membrane surface area comparable to that obtained following the insertion of fatty acid micelles^29^ (Fig. 3B). Similar results were obtained with 1-palmitoyl-2-oleoyl-*sn-glycero*-3-phosphocholine (POPC) vesicles (Fig. S28†). These results show that 2′DA3′P and *N*-DecArg embed into vesicle membranes and induce their growth. While the direct localisation of (oligo)nucleotides to membranes can be achieved *via* lipidated derivatives, synthetic peptides bearing hydrophobic and cationic moieties have also been shown to interact with both membranes and RNA and enable their co-localisation.^25,28^ We therefore evaluated the ability of *N*-DecArg to electrostatically interact with RNA and induce its recruitment to the vesicle membrane. We prepared giant vesicles made of model (OA and POPC) and prebiotic^15^ (monodecanoyl glycerol-1,2-cyclic phosphate (MDGCP) with DOH, in a 2 : 1 ratio) lipids as previously reported.^25^ When *N*-DecArg and a fluorescently tagged 7-mer RNA strand were added to the vesicle solution, and the mixture was incubated for 30 min at room temperature, confocal microscopy of both the model and prebiotic vesicle solutions confirmed that RNA binds to the outer surface of the lipid bilayer without penetrating the membrane or affecting vesicle stability (Fig. 3C and S29†). In control experiments omitting *N*-DecArg, no such localisation was observed (Fig. 3C and S29†).

The activation (MeNC) of nucleotides and amino acids in the presence of DA-based vesicles yields lipidated biomolecules, thus opening up a range of possibilities for exploring transient lipophilic derivatives of nucleic acids and peptides that anchor to the lipid bilayer and drive selective processes which could have been advantageous for the evolution of more specialised primitive cells. Moreover, the moderate stability of the 2′-decanoylated derivative of A3′P (*t*_1/2_ ∼ 5 days) suggests that similar lipid-(oligo)nucleotide adducts could have enabled the transient localisation of nucleic acid strands to primitive membranes without permanently sequestering them or preventing their participation in non-enzymatic elongation processes.

### Activation chemistry supports the selection of cyclophospholipids

**Fig. 4:**
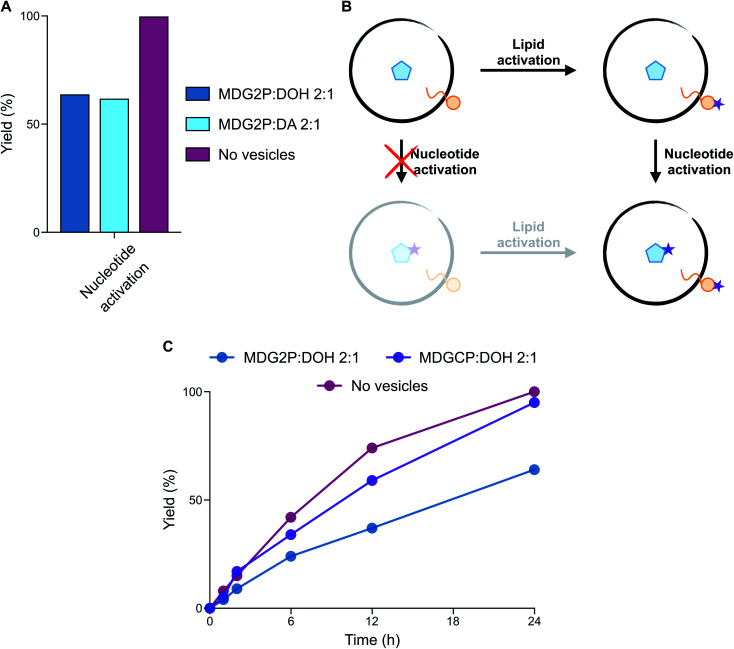
Evaluation of the compatibility of phospholipid-based vesicles and activation chemistry. (A) Plot reporting the yield (%) of nucleotide (A3′P) activation in the absence and presence of vesicles composed of MDG2P after 24 h in DCI buffer, pH 6. (B) Schematic representation of the two potential reaction pathways leading to the activation of both lipids and nucleotides. The preferential pathway describes the selectivity of lipid activation, followed by nucleotide activation. (C) Plot representing the yield (%) of nucleotide (A3′P) activation in the presence of phospholipid-based vesicles in DCI buffer, pH 6. The profile of nucleotide activation in the absence of vesicles (dark pink line) is reported for comparison. Plotted data are representative of ^31^P-NMR experiments.

We have recently shown that monoacylglycerol phosphates result from the activation of fatty acid carboxylates in the presence of glycerol-2-phosphate.^15^ However, these monoacylglycerol phosphates form stable vesicles only in combination with other lipids, such as diacylglycerol phosphates.^21^ Since mixtures of amphiphiles, including fatty acids, aldehydes, and alcohols, were likely present on early Earth, we evaluated the self-assembly of monodecanoylglycerol-2-phosphate (MDG2P) in combination with other prebiotic amphiphiles. Using the colorimetric assay based on merocyanine 540 described above and confocal microscopy, we observed the formation of vesicles when MDG2P was mixed with DOH or DA in a 2 : 1 ratio (Fig. S30 and S31†). Like fatty acids and peptides, acylglycerol phosphates and nucleotides share a common functional group and can competitively undergo activation. We thus investigated whether nucleotide phosphate activation could selectively occur in the presence of monoacylglycerol phosphate-based vesicles. Activation of 10 mM A3′P with MeNC in DCI buffer at pH 6 gave A > P in quantitative yield within 24 h (Fig. 2C). However, in the presence of 15 mM MDG2P : DOH (2 : 1 ratio) vesicles A > P was only generated in 64% yield after 24 h (Fig. 4A, S32 and S33†), while MDG2P was quantitatively converted into its cyclic phosphate derivative, MDGCP.

Since phospholipid-based vesicles are stable over a wider range of pH values than fatty acid-based vesicles, we repeated the same reaction in the optimal conditions for activation chemistry (pH 4). The acid-catalysed^15^ cyclisation of A3′P proceeded more rapidly, but the selectivity of the reaction remained unchanged such that A > P was formed in 62% yield after 6 h and MDG2P was again quantitatively converted to MDGCP. Complete cyclisation of the nucleotide was, however, observed after 24 h (Fig. S34†). Given the equal concentrations of MDG2P and A3′P in these reactions, our results show that cyclisation of the lipid phosphate in the membrane occurs more rapidly than that of the freely diffusing nucleotide phosphate (Fig. 4B). To determine if the lipid bilayer plays a role in the enhanced reactivity of MDG2P over A3′P, a competition reaction was set up in which activation of a 1 : 1 mixture of MDG2P and A3′P was performed in 1 : 1 water : acetonitrile to prevent the formation of any lipid supramolecular structure. Cyclisation of both substrates took place at comparable rates and complete conversion of each substrate was observed within 96 h (Fig. S35†). Thus, supramolecular assembly leads to an increase in the rate of cyclisation of the phosphate headgroups of amphiphiles. The exact nature of this effect is the subject of ongoing investigations.

Acylglycerol cyclic phosphates (cyclophospholipids) are minor products of the same activation chemistry that yields mostly monoacylglycerol phosphates from fatty acids and glycerol 2-phosphate under paste-like conditions or in formamide-rich solutions.^8,15^ Previous work demonstrated that cyclophospholipids are attractive from a prebiotic perspective because of their remarkable ability to self-assemble in a wide range of pH values and salt concentrations.^30^ Therefore, the prebiotic conversion of acylglycerol phosphates into their cyclic derivatives, as described above, provides a new high-yielding pathway for their formation and thus supports their proposed role in the emergence of primitive cells. When a solution containing A3′P and MDGCP : DOH (2 : 1 ratio) vesicles was exposed to MeNC, A > P formed rapidly at both pH 4 and 6 (Fig. 4C and S36†), without affecting the stability of the vesicles (Fig. S3†). Activation of A3′P with MeNC and acetaldehyde under Passerini conditions (imidazole buffer, pH 6.5) also gave A > P in quantitative yield after 30 min in the presence of MDGCP and DOH (2 : 1 ratio) vesicles (Fig. S37†). We next turned our attention to the reactivity of A5′P, due to its importance in non-enzymatic RNA polymerisation.^13^ Upon reaction with MeNC and acetaldehyde under Passerini conditions, 85% of the corresponding adenosine 5′-phosphorimidazolide was formed after 30 min in the presence of MDGCP-based vesicles (Fig. S38†). The yield of this reaction was comparable to that obtained in the control reaction performed in the absence of vesicles. These results suggest that cyclophospholipid-based vesicles could have potentially tolerated a range of different activation chemistries.

### Activation chemistry links nucleotides and peptides within primitive cells

The abiotic synthesis of peptidyl-RNA adducts was likely fundamental to the emergence of coded peptide synthesis and the advent of primordial translation machinery. When peptides were activated together with nucleotides, a mixture of longer peptides, nucleotide cyclic phosphates, and peptidyl nucleotides were obtained.^15^ Since the simultaneous activation of amino acids or peptides and nucleotides has not yet been studied in the presence of vesicles, we subjected mixtures of A3′P, protected amino acids and a range of prebiotic vesicles to activation conditions.

**Fig. 5:**
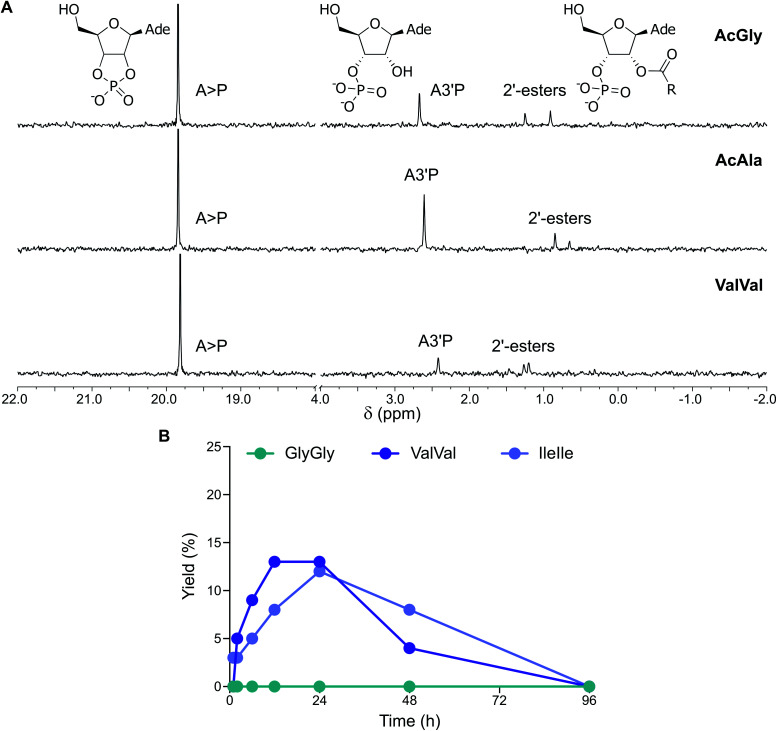
Simultaneous activation of nucleotides and peptides in the presence of vesicles made of prebiotic fatty acids. (A) ^31^P-NMR spectra showing the activation of A3′P in the presence of AcGly, AcAla and ValVal and DA : DOH : DHO (4 : 1 : 1 ratio) vesicles after 12 h in DCI buffer, pH 6. (B) Plot reporting the formation and the hydrolysis of 2′-esters of A3′P under activation conditions in the presence of the dipeptides GlyGly, ValVal or IleIle and DA : DOH : DHO (4 : 1 : 1 ratio) vesicles in DCI buffer, pH 6.

When *N*-acetylglycine (AcGly) and A3′P were simultaneously activated with MeNC, either in the presence or absence of fatty acid-based vesicles (DA : DOH : DHO 4 : 1 : 1 ratio, pH 6) in DCI buffer at pH 6, complete cyclisation of the nucleotide to A > P was observed after 96 h (Fig. S39†). In addition, the activation of the peptide carboxylate, its trapping by the 3′-phosphate, and intramolecular transfer to the 2′-hydroxyl of the nucleotide resulted in a transient peptidylated nucleotide, detectable by ^31^P-NMR spectroscopy (Fig. 5A and S40†). This 2′-peptidylated nucleotide is susceptible to hydrolysis and ultimately A > P is the final product. In the presence of vesicles, the 2′-peptidylated nucleotide accumulated for up to 6 h and a maximum yield of 13% was attained, comparable to that observed in the control reaction performed in the absence of vesicles (11%). Similar results were also observed with *N*-acetylalanine (AcAla) (Fig. 5A and S41†). Interestingly, when the activation (MeNC) chemistry was performed using a mixture of A5′P and AcGly or AcAla in the presence of DA : DOH : DHO (4 : 1 : 1 ratio, pH 6) vesicles, approximately 5% of the corresponding adenosine 5′-mixed anhydride was detected after 12 h (Fig. S42 and S43†). The identity of this species was confirmed by comparison of its NMR spectroscopic data with that previously reported for the equivalent reaction performed in the absence of vesicles.^15^

We then explored the activation of mixtures of A3′P and unprotected dipeptides, including GlyGly, AlaAla, IleIle and ValVal (Fig. S44†). In the presence of vesicles, the reactions of A3′P with GlyGly and AlaAla gave no peptidylated nucleotides and only A > P could be detected by ^1^H and ^31^P-NMR spectroscopy at time points up to 96 h (Fig. 5B, S45 and S46†). However, 12% and 13% 2′-peptidylated nucleotides were observed to have accumulated after 24 h when IleIle and ValVal were used, respectively (Fig. 5B). Similar results were obtained in the absence of vesicles (Fig. S47 and S48†). These findings demonstrate that 2′-peptidylated nucleotides deriving from hydrophobic peptides, which have the potential to interact with and localise to primordial membranes,^25^ accumulate to a greater extent than those derived from more hydrophilic peptides.

**Fig. 6:**
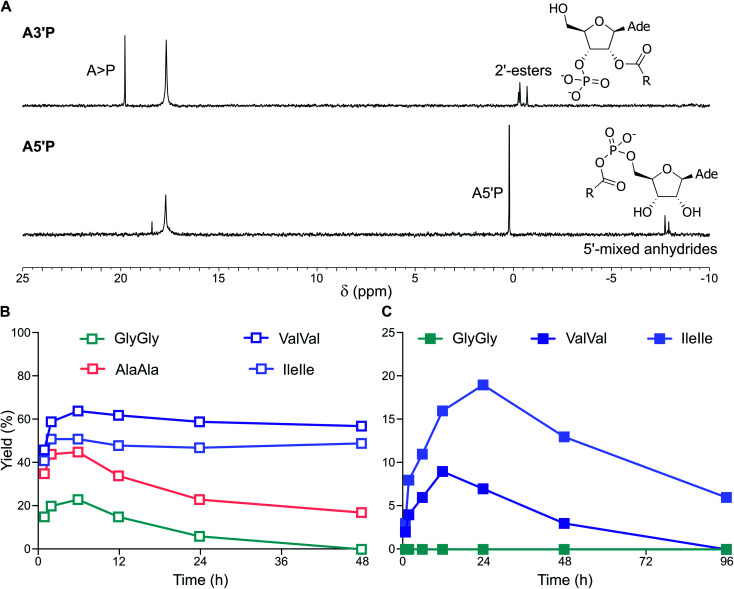
Simultaneous activation of nucleotides and peptides in the presence of vesicles made of prebiotic phospholipids. (A) ^31^P-NMR spectra showing the activation of A3′P and A5′P in the presence of ValVal and MDGCP : DOH (2 : 1 ratio) vesicles after 24 h in DCI buffer, pH 4. (B) Plot reporting the formation and the hydrolysis of 2′-esters of A3′P under activation conditions in the presence of the dipeptides GlyGly, AlaAla, ValVal or IleIle and MDGCP : DOH (2 : 1 ratio) vesicles in DCI buffer, pH 4. (C) Plot reporting the formation and the hydrolysis of 2′-esters of A3′P under activation conditions in the presence of the dipeptides GlyGly, ValVal or IleIle and MDGCP : DOH (2 : 1 ratio) vesicles in DCI buffer, pH 6.

Finally, we tested the simultaneous activation of both nucleotides and dipeptides in the presence of cyclophospholipid-based vesicles (MDGCP : DOH 2 : 1 ratio) in DCI buffer at pH 4 and 6. Activation of ValVal alongside A3′P or A5′P gave 2′-esters and 5′-mixed anhydrides, respectively, in both the presence or absence of vesicles (Fig. 6A and S49†). For example, the MeNC-mediated reactions of A3′P with ValVal at pH 4 and 6 were unaffected by the presence of vesicles and gave 62% and 9% peptidyl species, respectively, after 12 h, together with A > P (Fig. 6B and C). Overall, our results suggest that a prebiotically plausible scenario, in which nucleotides and peptides are joined *via* activation chemistry in the presence of vesicles, could have potentially supported the emergence of RNA-peptide systems compartmentalised within protocells on early Earth.

## Conclusions

Functional protocells could only have emerged and evolved if prebiotic chemical processes and supramolecular containers were mutually compatible. Here we demonstrate that activation chemistry selects for protocells compatible with the conditions required for the simultaneous emergence of oligonucleotides, peptides and peptidylated nucleotides. DA- and MDGCP-based vesicles can sustain the activation of nucleotides, amino acids and peptides with no apparent alteration or destabilisation. Intriguingly, we show that, upon activation, supramolecular assembly increases the rate of conversion of the monoacylglycerol phosphate precursor MDG2P into the cyclophospholipid MDGCP. Our results highlight the stability of these cyclophospholipids^30^ under the activation conditions essential for the conjugation of biomolecules and the subsequent emergence of functional protocells. This study therefore emphasises their suitability as robust constituents of protocell membranes. In parallel, we demonstrate for the first time that mixtures of simpler fatty acids, alcohols and aldehydes spontaneously self-assemble under acidic conditions. Vesicles formed of these components would have provided a reservoir of reactive monomers to give, upon combination with prebiotic building blocks, functional anchors, including lipidated nucleotides and peptides, that enable the localisation of nucleic acids and peptides to membranes.

Our study explores the potential early coevolution of cell membranes, nucleic acids and proteins through the investigation of complex mixtures of activated prebiotic building blocks, thus advancing along the trajectory that connects prebiotic chemistry to modern biology.

## Conflicts of interest

There are no conflicts to declare.

## Supplementary Material

SC-011-D0SC04506C-s001
