# Supplementary material for: Activation chemistry drives the emergence of functionalised protocells
Source: Chem Sci. 2020 Oct 2;11(39):10688–97. doi: 10.1039/d0sc04506c (PMC8162433; doi:10.1039/d0sc04506c)
Supplement: SC-011-D0SC04506C-s001 [file SC-011-D0SC04506C-s001.pdf]

Electronic Supplementary Material (ESI) for Chemical Science.
This journal is © The Royal Society of Chemistry 2020

Supporting information

Activation chemistry drives the emergence of functionalised protocells

Claudia Bonfio[§,*], David A. Russell[§], Nicholas J. Green, Angelica Mariani and John D. Sutherland

Medical Research Council Laboratory of Molecular Biology, Cambridge Biomedical Campus, Francis Crick Avenue, Cambridge CB2 0QH, United Kingdom

* Correspondence: bonfio@mrc-lmb.cam.ac.uk

Materials and methods

Supporting Figures 1 to 49

Supporting References

Materials and methods

Reagents and solvents were bought from Nu-Chek Prep, Avanti Polar Lipids, Sigma-Aldrich, VWR International, Alfa-Aesar and Acros Organics and were used without further purification unless otherwise stated. Oligonucleotides were purchased from IDT. For membrane growth studies, the phospholipids *N*-(7-nitrobenz-2-oxa-1,3-diazol-4-yl)-1,2-dihexadecanoyl-*sn*-glycero-3-phosphoethanolamine (NBD PE) and Lissamine rhodamine B 1,2-dihexadecanoyl-sn-glycero-3-phosphoethanolamine (Rh-DHPE) were used (Life Technologies). A Mettler Toledo SevenEasy pH Meter S20 was used to monitor the pH of the solutions, adjusted with either NaOH or HCl solutions as appropriate. Deoxygenation of H_2O:D_2O 9:1 mixtures was achieved by sparging anhydrous argon through the solution for 30 min. All reactions were carried out at room temperature unless otherwise stated. Purification of the synthesised molecules was performed by reverse-phase chromatography using a preparative Varian Prostar HPLC System equipped with an Atlantis T3 C18 Prep Column OBD 10 µm (19× 250 mm). Analytical HPLC was used for hydrolysis experiments, using a Waters Atlantis T3 C18 column (5 µm, 4.6 mm x 150 mm). All unknown compounds in the reaction mixtures were confirmed by comparison of NMR spectral data or spiking experiments with authentic compounds either purchased from Sigma-Aldrich or synthesised in house using conventional synthetic chemistry. ^{1}H, ^{31}P, and ^{13}C-NMR spectra were acquired using a Bruker Ultrashield 400 Plus operating at 400.1, 162.0, and 100.6 MHz respectively. Samples consisting of H_2O/D_2O mixtures were analysed using HOD suppression to collect ^{1}H-NMR data. Chemical shifts (δ) are given in ppm. Coupling constants (*J*) are given in Hertz (Hz) and the abbreviations s, d, t, m, and bs indicate singlet, doublet, triplet, multiplet, and broad singlet signals, respectively. The yields of conversion were determined by relative integration of the signals in the ^{1}H or ^{31}P-NMR spectra. Data analysis was performed using MestReNova (version 7.0) and GraphPad Prism (version 7.0b). For the synthesis of phospholipids, reactions were monitored by thin-layer chromatography (TLC) using silica gel coated aluminum plates 60 F254 (Merck) and spots were visualised by staining with $KMNO_4$ solution. Isolated yields were calculated following compounds purification. Direct phase chromatography was performed using Davisil silica gel (40 – 63 µm, Grace). Reverse phase chromatography was performed using Sep-Pak C18 Cartidges (Waters). UV-vis. analyses were performed on a Varian Cary 6000i UV/Vis/NIR spectrophotometer and fluorescence measurements were recorded on a Varian Cary Eclipse fluorescence spectrophotometer, both equipped with a multi-sample Peltier temperature controller. Mass spectra were recorded with an Agilent Technologies 6130 Quadrupole LC- MS using positive and negative Electron Spray Ionisation. Vesicles were purified by size exclusion chromatography with PD miniTrap G-10 columns, GE Healthcare, Sephadex G-10 Medium. Vesicles were imaged on a Zeiss LSM 780 confocal microscope equipped with a ×63 oil objective. Images were processed with Fiji[1]. All data shown are representative of distinct samples, n = 3 replicates. The syntheses of monodecanoyl glycerol-1,2-cyclic phosphate and *N*-decanoyl arginine were performed according to previously reported procedures[2–4].

Abbreviations. The following abbreviations are used throughout: DA, decanoic acid; DCI, 4,5-dicyanoimidazole; DHO, decanal; DOH, decanol; GMD, glycerol monodecanoate; MDGCP, monodecanoyl glycerol-1,2-cyclic phosphate; MDG2P, monodecanoyl glycerol-2-phosphate; OA, oleic acid; POPC, 1-palmitoyl-2-oleoyl-*sn*-glycero-3-phosphocholine; A>P, adenosine 2′,3′-cyclic phosphate; A3′P, adenosine 3′-monophosphate; 2′-AA3′P, 2′-acetyl adenosine 3′-monophosphate 2′-DA3′P, 2′-decanoyl adenosine 3′-monophosphate.

General procedure for the activation of carboxylates and phosphates. A solution of 10 mM A3′P alone or in combination with 100 mM carboxylates (N-protected amino acids or non-protected peptides) in 100 mM DCI buffer (0.5 mL, H_2O:D_2O 9:1) was prepared and the pH was

adjusted to the required value with HCl (1.0 M) and NaOH (1.0 M) solutions. The solution was then used for the preparation of vesicles as previously reported. 10.4 μL methyl isocyanide (final concentration: 400 mM) was then added and the reaction was monitored by ^{1}H- and ^{31}P-NMR spectroscopy over the course of 4 d.

Preparation of vesicles. Vesicles made of fatty acid mixtures or acylglycerol phosphates were prepared by direct dispersion in an aqueous buffered solution as previously reported[5]. Unless otherwise stated, a total lipid concentration of 25 mM and 15 mM was used for fatty acid- and phospholipid-based vesicles, respectively. Samples were briefly vortexed, tumbled at room temperature for 30 min and then extruded with 11 passages through a 100 nm pore membrane (Whatman) using a Mini-Extruder (Avanti Polar Lipids).

For microscopy, giant unilamellar vesicles were prepared by thin film hydration methods[6] on the bottom of glass vials. Vesicles, prepared from POPC or oleic acid, contained 0.15 mol % Liss Rh-PE or NBD-PE. Vesicles made of MDGCP:DOH 2:1 were stained with Nile Red dye. Vesicle samples with *N*-decanoyl-arginine were prepared by premixing POPC or OA with different amounts of *N*-decanoyl-arginine and 0.15 mol % Liss Rh-PE or NBD-PE in chloroform before being dried on the surface of glass vials. Vesicles made of MDGCP:DOH 2:1 were prepared by premixing the lipid mixture with different amounts of *N*-decanoyl-arginine in water before being dried on the surface of glass vials. Solvent was evaporated for >12 h, and lipid films were hydrated with sucrose + Tris solution (200 mM sucrose +100 mM Tris-HCl buffer, pH 8.5) and left overnight in the dark at room temperature to form giant unilamellar vesicles.

Vesicle formation and critical vesicle concentration (CVC) determination. The formation and the CVC of vesicles made of fatty acid or acylglycerol phosphate mixtures in aqueous buffered solution was determined using merocyanine 540 as a solvatochromic probe, following changes in the absorbance ratio $A_{565\ nm}/A_{525\ nm}$. 0.5 μL merocyanine 540 solution was added to a serial dilution of the samples (500 μL) from a 2.5 mM stock solution in ethanol. The diluted samples were left to equilibrate in the dark for half an hour before measurements were made.

Vesicle stability studies under activation conditions. Vesicles made of DA:DOH:DHO 4:1:1 or MDGCP:DOH 2:1 (total lipid concentration 25 mM) were prepared in 100 mM DCl buffer (pH 6 and 4, respectively). 10.4 μL methyl isocyanide alone or in combination with 2.8 μL acetaldehyde (final concentrations: 400 mM and 100 mM, respectively) was then added. The samples were analysed by UV-vis. spectrophotometry in the presence of merocyanine 540 as previously described[19]. For microscopy analyses, 5 µl droplets of vesicles were deposited on untreated glass slides.

Membrane growth assay. POPC and OA vesicles were prepared with 0.1 mol % of the FRET dyes NBD PE and Liss Rh-PE. Growth from the addition of oleate micelles, N-decanoylated arginine or 2′-decanoylated-A3′P was assessed by adding each reagent to a vesicle solution (1, 2, and 4 mM) and monitoring the resulting change in FRET signal as previously described[27]. *N*-decanoyl-arginine and 2'-decanoyl-A3′P were dissolved in water and oleate micelles were prepared by dissolving oleic acid in 500 mM NaOH (final oleate concentration: 20 mM). FRET was measured using the fluorescence ratio between the donor (λ_{em} 517 nm) and the acceptor (λ_{em} 590 nm) lipids, with an excitation at 463 nm. The FRET signal was converted into relative surface area through a standard curve correlating mol % of FRET dyes in the membrane to FRET signal. The change in surface area after incubation for 10 h with each reagent was reported.

RNA localisation studies. For studies on the RNA localisation to the outside of vesicle membranes with N-decanoylated arginine, vesicle membranes containing 10, 25, and 50 mol % N-decanoylated arginine were mixed with RNA, so that the final concentration of each component was 100 µM vesicles, 10, 25, or 50 µM N-decanoylated arginine, and 2 µM RNA for studies with POPC vesicles and 10 mM vesicles, 1, 2.5, or 5 mM N-decanoylated arginine, and 10 µM RNA for studies with OA and MDGCP:DOH 2:1 vesicles. After incubation for 30 min, 15 µL of the vesicle/peptide/RNA mixture was diluted 20-times into either 125 mM Tris–HCl, 500 µM oleic acid or 500 µM MDGCP:DOH 2:1 mixture and imaged. The RNA used in these studies was 5′-(FAM)-GGT-TCT-C-3′ (HPLC Purified).

Chemical synthesis of 2′-decanoyl adenosine 3′-monophosphate and hydrolysis studies. A solution of 1-decanoylimidazole (8.9 mg, 0.04 mmol, 2.0 equiv.) in MeCN (3.0 mL) was added to a solution of A3′P (sodium salt) (7 mg, 0.02 mmol, 1.0 equiv.) in D_2O (1.0 mL) at pH 7. The reaction was monitored by ^{31}P-NMR spectroscopy After 4 days at room temperature the solvent was evaporated to give a colourless oily residue. This material was purified by preparative reverse-phase HPLC (eluent A: 20 mM NH_4OAc buffer, pH 4.5, eluent B: MeCN, gradient 95:5 to 30:70). 2′-Decanoyl adenosine 3′-monophosphate was obtained as a white solid (6.2 mg, 59% yield based on the mass of the monoammonium salt) and characterised by ^{1}H, ^{13}C and ^{31}P-NMR spectroscopy. The hydrolysis studies were performed by analytical HPLC (eluent A: 20 mM NH_4OAc buffer, pH 4.5, eluent B: MeCN, gradient 95:5 to 30:70).

^{1}H-NMR (400 MHz, D_2O) δ 8.36 (s, 1H), 8.21 (s, 1H), 6.30 (d, *J* = 7.7 Hz, 1H), 5.69 (m, 1H), 3.88 (t, *J* = 3.0 Hz, 2H), 3.15 (q, *J* = 7.4 Hz, 6H), 2.43 (m, 1H), 2.31 (dd, *J* = 14.7, 7.4 Hz, 1H), 2.17 (s, 0.5H), 1.43 (m, 2H), 1.23 (t, *J* = 7.3 Hz, 12.5H), 0.91 (m, 16H);^{13}C-NMR (100 MHz, D_2O) δ 174.5, 153.8, 150.3, 148.4, 141.2, 118.7, 86.2, 85.7, 74.1, 72.6, 61.2, 33.4, 31.2, 28.7, 28.5, 28.4, 28.1, 24.1, 22.1, 13.4; ^{31}P-NMR (100 MHz, D_2O) δ 0.10.

Chemical synthesis of 2′-acetyl adenosine 3′-monophosphate and hydrolysis studies. 1-Acetylimidazole (14 mg, 0.127 mmol, 2.2 equiv.) was added to a solution of A3′P (sodium salt) (20 mg, 0.058 mmol, 1.0 equiv.) in D_2O (1.0 mL). The reaction was monitored by ^{1}H-NMR spectroscopy. After 2 hours at room temperature, the solution was cooled and kept at 4°C for 64 hours. 10 mM ammonium acetate buffer (pH 4.5) was then added to a final volume of 5.0 mL and the material was purified by preparative reverse phase HPLC (eluent A: 20 mM NH_4OAc buffer, pH 4.5, eluent B: MeCN, gradient 95:5 to 30:70). 2′-Acetyl adenosine 3′-monophosphate was obtained as a white solid (3.5 mg, 15% yield based on the mass of the monoammonium salt) and characterised by ^{1}H, ^{13}C and ^{31}P-NMR spectroscopy. The hydrolysis studies were performed by analytical HPLC (eluent A: 20 mM NH_4OAc buffer, pH 4.5, eluent B: MeCN, gradient 95:5 to 30:70).

^{1}H-NMR (400 MHz, D_2O) δ 8.37 (s, 1H), 8.26 (d, *J* = 0.8 Hz, 1H), 6.33 (d, *J* = 6.1 Hz, 1H), 5.75 (t, *J* = 5.8 Hz, 1H), 5.00 (ddd, *J* = 8.8, 5.4, 3.5 Hz, 1H), 4.53 (q, *J* = 3.2 Hz, 1H), 3.96 (qd, *J* = 13.0, 2.9 Hz, 2H), 2.15 (s, 3H). ^{13}C-NMR (100 MHz, D_2O) δ 170.7, 153.7, 150.6, 146.5, 138.7, 117.1, 84.3, 83.6, 72.3, 70.3, 59.2, 18.0. ^{31}P-NMR (100 MHz, D_2O) δ 1.67.

Formation of *N*-methyl guanidylated (oligo)arginine and *N*-decanoyl-(oligo)arginine under activation conditions. An aqueous solution (0.5 mL, H_2O:D_2O 9:1) of 100 mM L-Arg and 100 mM DCl was adjusted to pH 6 and was used for the preparation of fatty acid-based vesicles. 10.4 µL methyl isonitrile (final concentration: 400 mM) was then added and the mixture was incubated at room temperature. The reaction was monitored periodically by ^{1}H- and ^{31}P-NMR spectroscopy and mass spectrometry.

General procedure for the synthesis of monodecanoyl glycerol-1,2-cyclic phosphate.

- Synthesis of solketal monodecanoate. To a solution of C_{10}-acid (5 mmol) in dry DCM (25 mL) was added a solution of DMAP (0.12 g, 1 mmol) in dry DMC (5 mL), followed by solketal (0.62 mL, 5 mmol). The mixture was cooled to 0 °C prior to the dropwise addition of a solution of DCC (1.03 g, 5 mmol) in dry DCM (20 mL). The reaction was stirred at RT overnight, after which the dicyclohexylurea was filtered. The reaction was washed twice with a $NaHCO_3$ saturated solution, and the aqueous phase was further extracted with DCM. The organic layer was dried over Na_2SO_4 and concentrated under reduced pressure. The crude product was purified by direct phase chromatography (hexane:ethyl acetate, 8:2).
  Solketal monodecanoate (colorless oil): ^{1}H-NMR (400.1 MHz, $CDCl_3$) δ 4.34-4.28 (m, 1H), 4.16 (dd, J = 11.5, 4.7 Hz, 1H), 4.11-4.06 (m, 2H), 3.74 (dd, J = 8.4, 6.2 Hz, 1H), 2.34 (t, J = 7.6 Hz, 2H), 1.62 (p, J = 7.3 Hz, 3H), 1.43 (s, 3H), 1.37 (s, 3H), 1.34-1.20 (m, 12H), 0.88 (t, J = 6.8 Hz, 3H).
- Synthesis of glycerol monodecanoate. A solution of solketal monodecanoate (4.1 mmol) in MeOH (135 mL) was cooled to 0 °C prior to the dropwise addition of a 1 N solution of HCl in water (2.9 mL). The reaction was stirred at RT for 7 hours. The reaction was quenched with a $NaHCO_3$ saturated solution and then extracted with DCM. The organic layer was dried over Na_2SO_4 and concentrated under reduced pressure. The crude product was purified by direct phase chromatography (ethyl acetate).
  Glycerol monodecanoate (white solid): ^{1}H-NMR (400.1 MHz, $CDCl_3$) δ 4.18 (qd, J = 11.7, 5.3 Hz, 2H), 3.96-3.90 (m, 1H), 3.72-3.68 (m, 1H), 3.63-3.59 (m, 1H), 2.35 (t, J = 7.6 Hz, 2H), 1.63 (p, J = 7.2 Hz, 2H), 1.37-1.21 (m, 12H), 0.88 (t, J = 6.6 Hz, 3H).
- Synthesis of monodecanoyl glycerol-1,2-cyclic phosphate. To a solution of $POCl_3$ (2.0 mL, 22.1 mmol) in dry THF (15 mL) at -78 °C, dry pyridine (1.8 mL, 22.1 mmol) was added dropwise under argon. The mixture was stirred at -78 °C for 5 min, after which a solution of glycerol monodecanoate (3 mmol) in dry THF (15 mL) was added to it dropwise (over 15 min). The reaction was stirred at -78 °C for 30 min, warmed to RT and additionally stirred for 2 hours. The reaction was then poured into an ice-cold solution of $NaHCO_3$ (10.3 g in 85 mL) and stirred for ~ 1 hour. The aqueous phase was washed with ethyl acetate and concentrated under reduced pressure to remove organic solvent residues. The sample was lyophilised and then redissolved in water:ACN (1:1, 60 mL). The insoluble phosphate salts were removed by filtration and the filtrate was concentrated under reduced pressure to remove organic solvent residues. The sample was desalted by reverse phase chromatography (gradient: water - water:ACN, 1:1).
  Monodecanoyl glycerol-1,2-cyclic phosphate (white solid): ^{1}H-NMR (400.1 MHz, D_2O) δ 4.77-4.71 (m, 1H), 4.43-4.34 (m, 2H), 4.26 (dd, J = 12.9, 6.0 Hz, 1H), 4.10 (td, J = 9.6, 6.4 Hz, 1H), 2.48 (t, J = 7.4 Hz, 2H), 1.66 (p, J = 6.8 Hz, 2H), 1.36-1.31 (m, 12H), 0.90 (t, J = 6.5 Hz, 9H). ^{31}P-NMR (162.0 MHz, D_2O, 1H-decoupled) δ 18.0. ^{13}C-NMR (100.6 MHz, D_2O) δ 176.6, 74.0 (d, JC-P = 2.8 Hz), 65.7, 64.2 (d, JC-P = 5.6 Hz), 33.6, 31.2, 28.6, 28.5, 28.4, 28.3, 24.2, 22.0, 13.5.

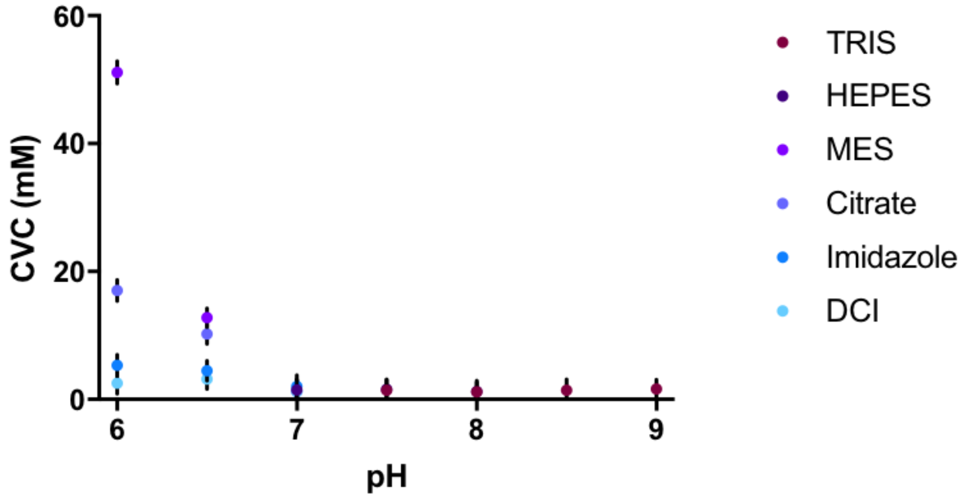

Figure S1 Determination of the critical vesicle concentration of vesicles made of DA:DOH:DHO 4:1:1 in different buffers, based on the merocyanine 540 assay[7]. Data are representative of three independent experiments and values are expressed as the mean ± SEM.

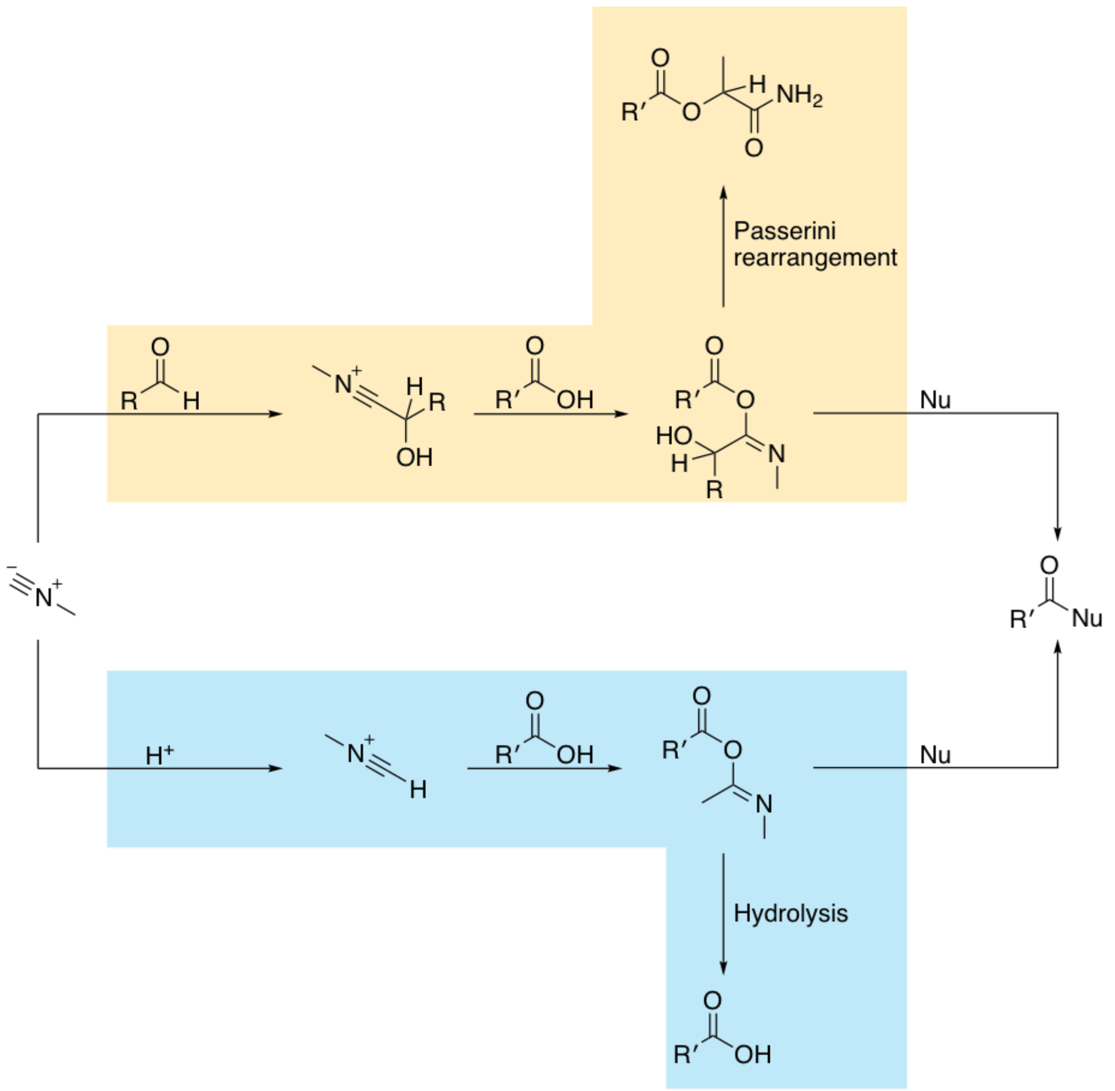

Figure S2 Reaction scheme showing isocyanide-driven carboxylate activation chemistries in the presence of either aldehydes (orange) or specific acid (blue)[8].

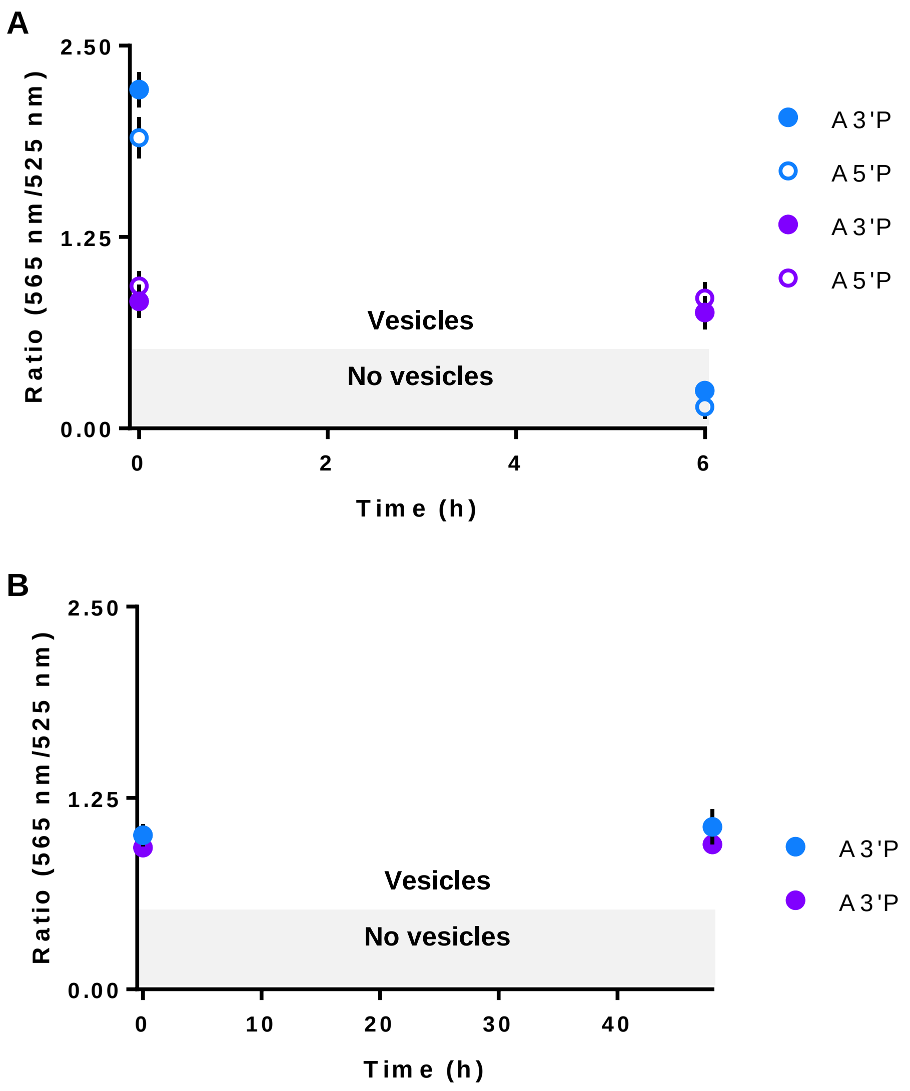

Figure S3 Survival of vesicles under activation chemistry conditions. (A) Survival of vesicles following the activation of 10 mM nucleotide using 400 mM methyl isocyanide and 100 mM acetaldehyde in 100 mM imidazole buffer at pH 6.5. Blue dots are representative of experiments performed with fatty acid-based vesicles (DA:DOH:DHO 4:1:1). Violet dots are representative of experiments performed with phospholipid-based vesicles (MDGCP:DOH 2:1). Experiments were performed on both A3'P (filled dots) and A5'P (empty dots). (B) Survival of vesicles following the activation of 10 mM A3'P using 400 mM methyl isocyanide in 100 mM DCI buffer at pH 6. Blue dots are representative of experiments performed with fatty acid-based vesicles (DA:DOH:DHO 4:1:1). Violet dots are representative of experiments performed with phospholipid-based vesicles (MDGCP:DOH 2:1). Data are representative of three independent experiments and values are expressed as the mean ± SEM.

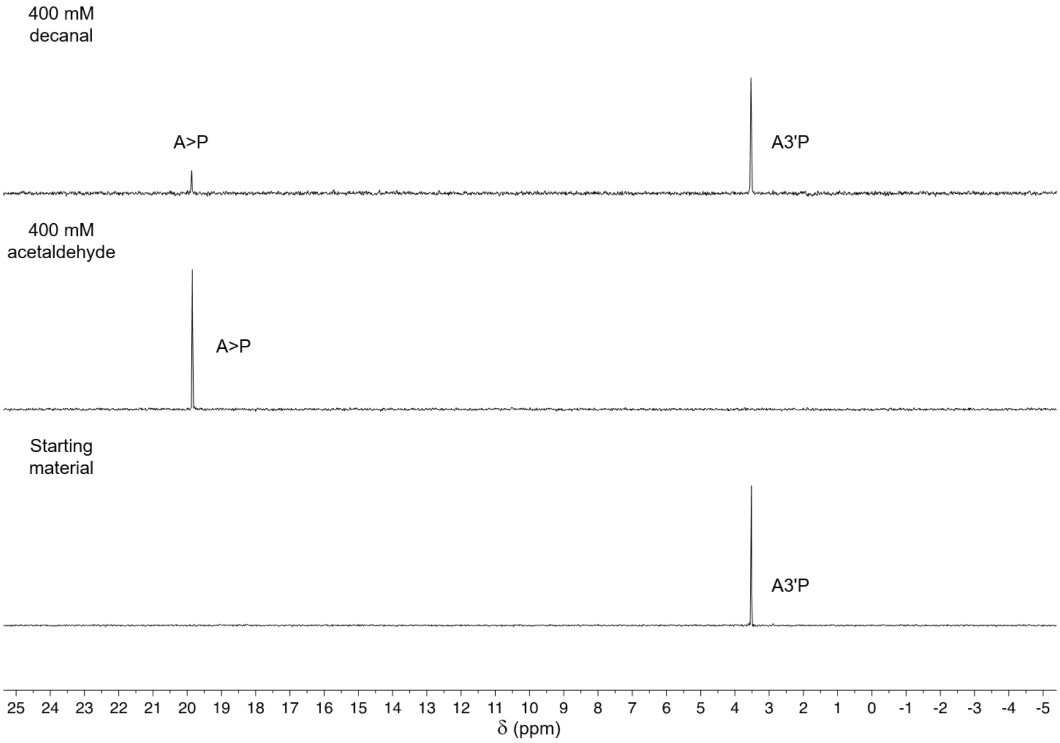

Figure S4 ^{31}P-NMR spectra showing the methyl isocyanide-driven activation of 10 mM A3'P after 2 h in the presence of 400 mM decanal (top) or 400 mM acetaldehyde (middle) in 100 mM imidazole buffer, pH 6.5. The starting material A3'P is reported (bottom) for comparison.

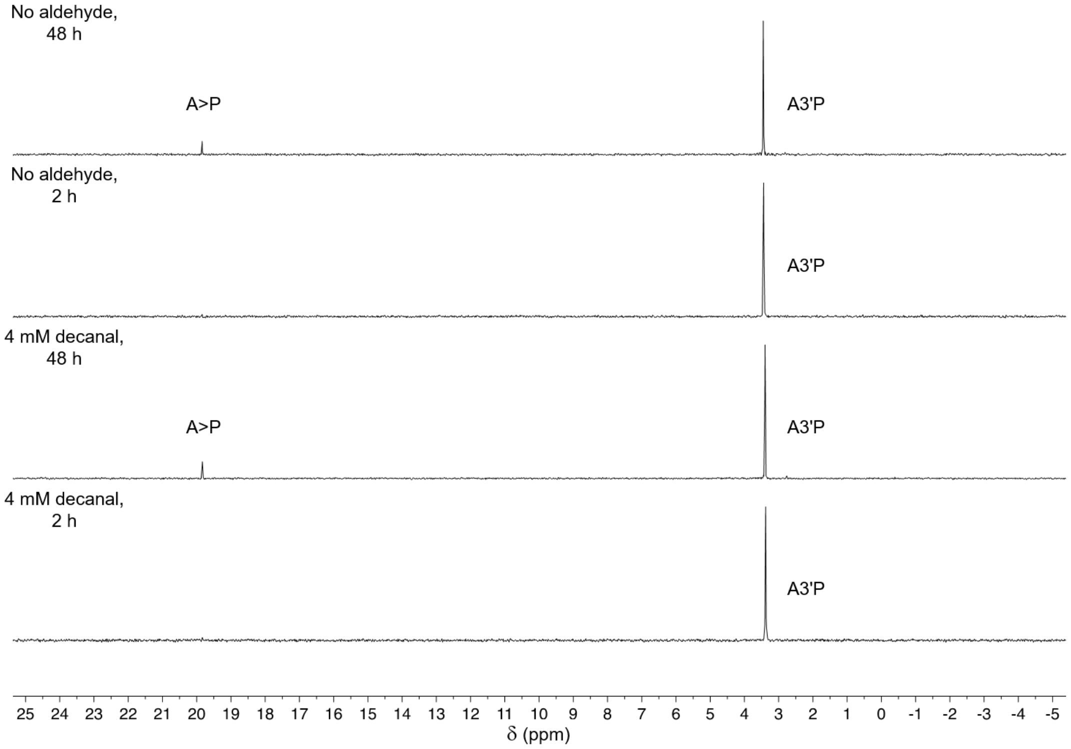

Figure S5 ^{31}P-NMR spectra showing the methyl isocyanide-driven activation of 10 mM A3'P in the presence or absence of 4 mM decanal in 100 mM imidazole buffer, pH 6.5. The starting material A3'P is reported (bottom) for comparison.

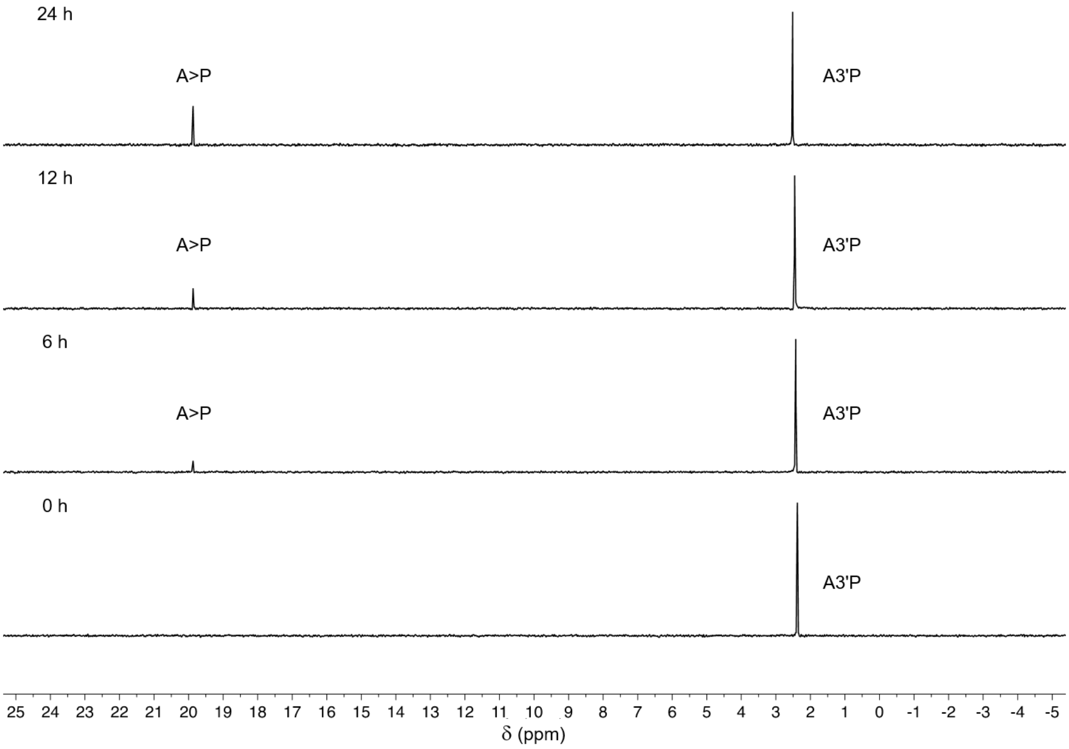

Figure S6 ^{31}P-NMR time course showing the activation of 10 mM A3’P in the presence of vesicles made of DA:DOH:DHO 4:1:1, 25 mM total lipid concentration, in 100 mM citrate buffer, pH 6.

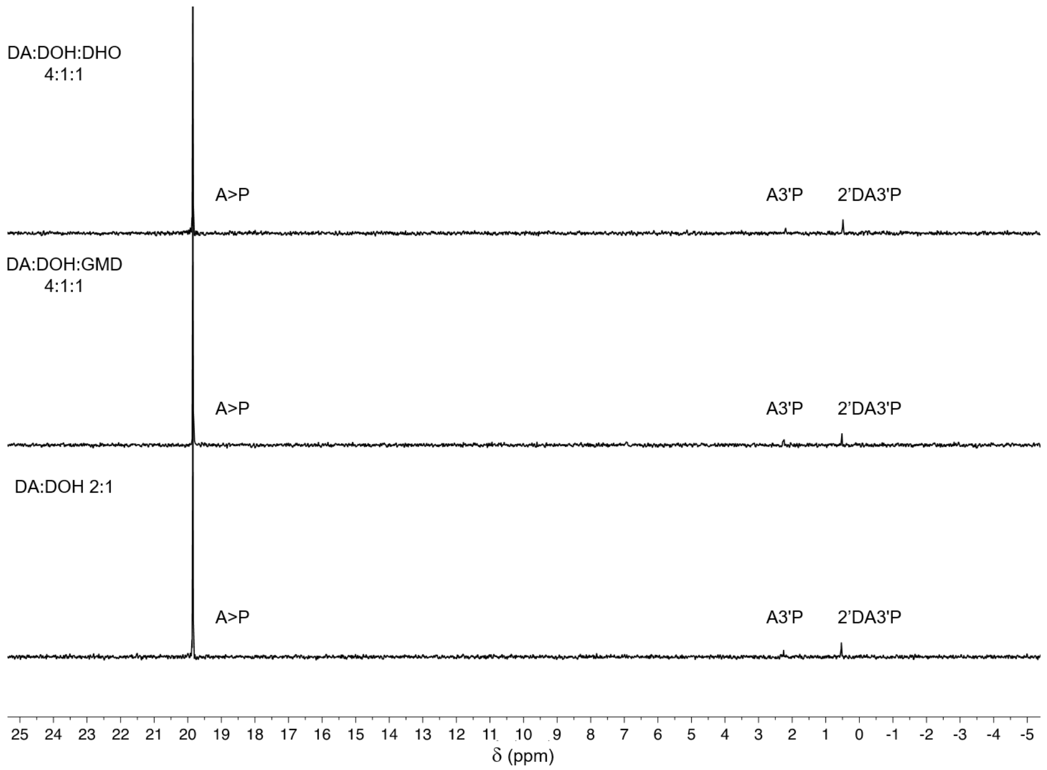

Figure S7 ^{31}P-NMR spectra showing the activation of 10 mM A3’P after 24 h in the presence of vesicles made of DA:DOH 2:1 (bottom), DA:DOH:GMD 4:1:1 (middle) or DA:DOH:DHO 4:1:1 (top), 25 mM total lipid concentration, in 100 mM DCI buffer, pH 6.

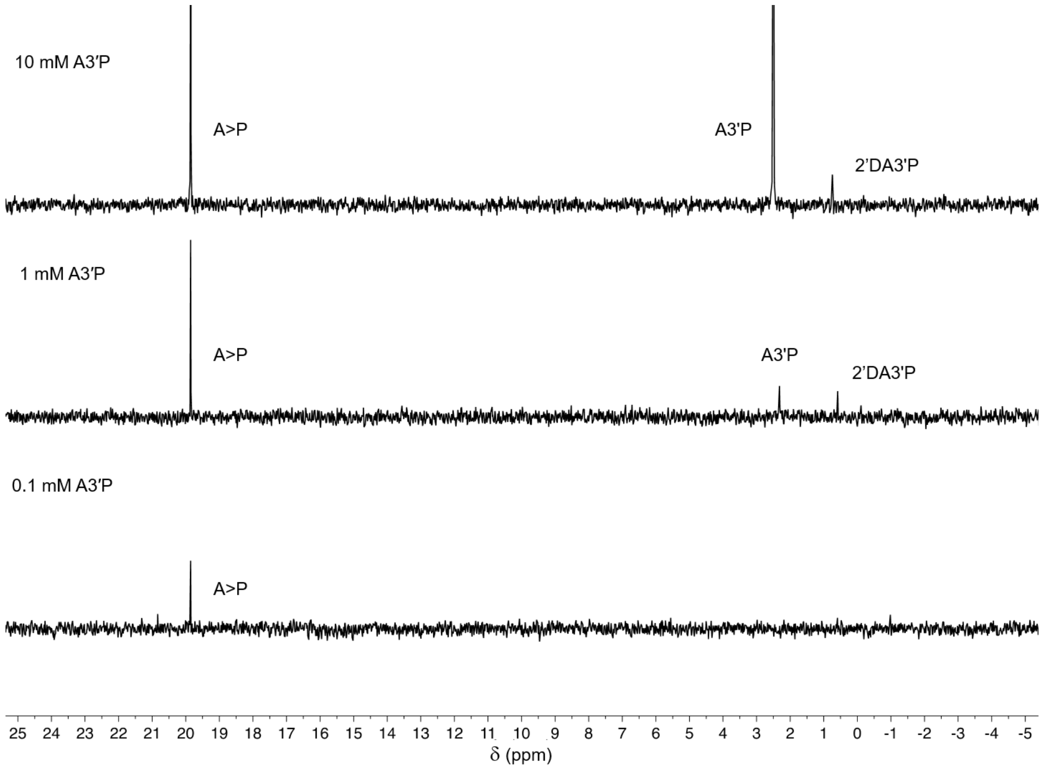

Figure S8 ^{31}P-NMR spectra showing the activation of 10 mM (top), 1 mM (middle) or 0.1 mM (bottom) A3′P after 12 h in the presence of vesicles made of DA:DOH:DHO 4:1:1, 25 mM total lipid concentration, in 100 mM DCI buffer, pH 6.

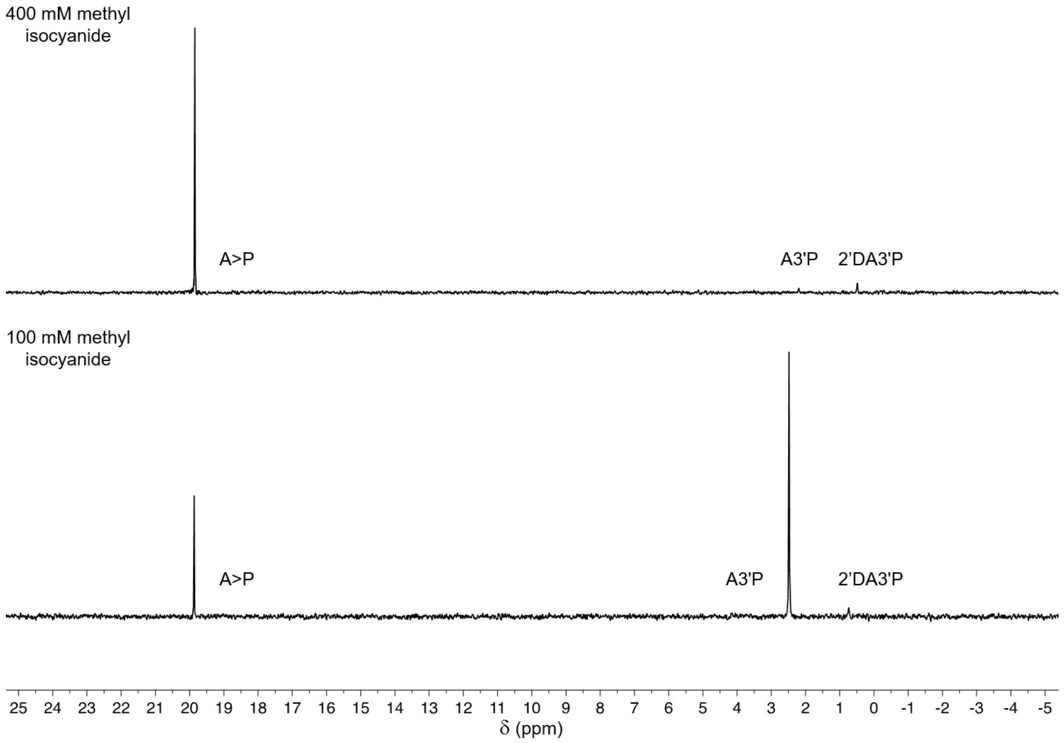

Figure S9 ^{31}P-NMR spectra showing the activation of 10 mM A3'P after 24 h with 400 mM (top) or 100 mM (bottom) methyl isocyanide in the presence of vesicles made of DA:DOH:DHO 4:1:1, 25 mM total lipid concentration, in 100 mM DCl buffer, pH 6.

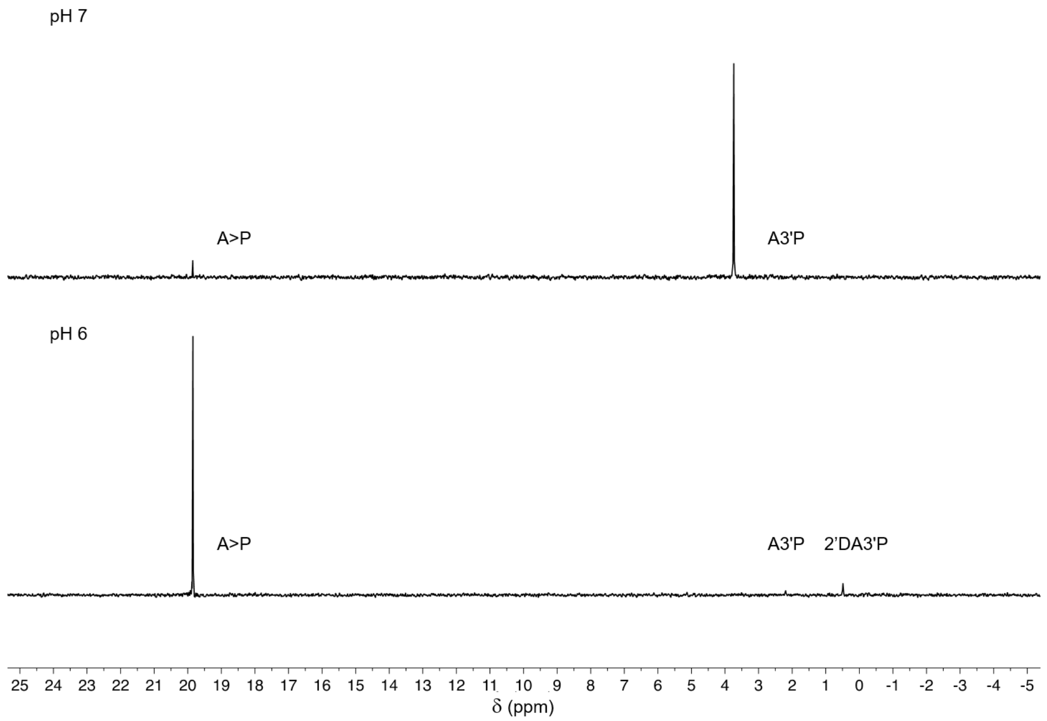

Figure S10 ^{31}P-NMR spectra showing the activation of 10 mM A3'P after 24 h in the presence of vesicles made of DA:DOH:DHO 4:1:1, 25 mM total lipid concentration, in 100 mM DCI buffer, pH 6 (bottom) and pH 7 (top).

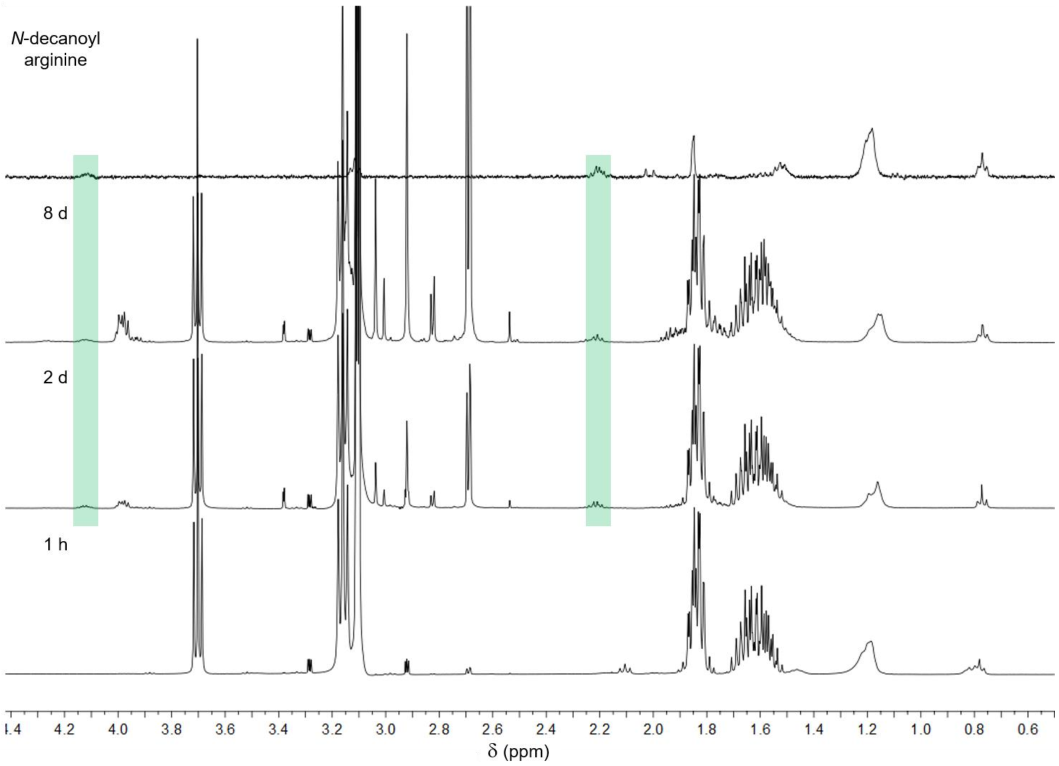

Figure S11 ^{1}H-NMR time course showing the formation of arginine oligomers and decanoylated adducts from the activation of 100 mM arginine in the presence of vesicles made of DA:DOH:DHO 4:1:1, 25 mM total lipid concentration, in 100 mM DCl buffer, pH 6. The NMR spectrum of *N*-decanoyl arginine in D_2O is reported for comparison (top). The characteristic signals for *N*-decanoyl arginine are highlighted in green.

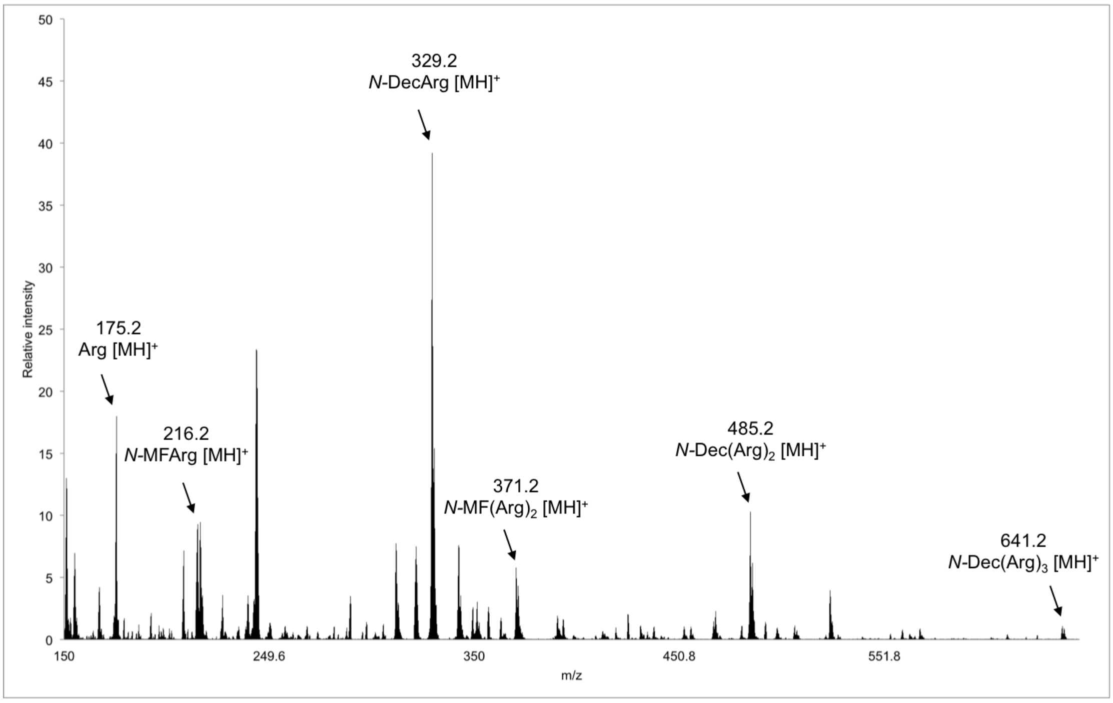

Figure S12 ESI-Mass spectrum of oligopeptide derivatives detected upon the activation of 100 mM arginine after 8 days in the presence of vesicles made of DA:DOH:DHO 4:1:1, 25 mM total lipid concentration, in 100 mM DCI buffer, pH 6.

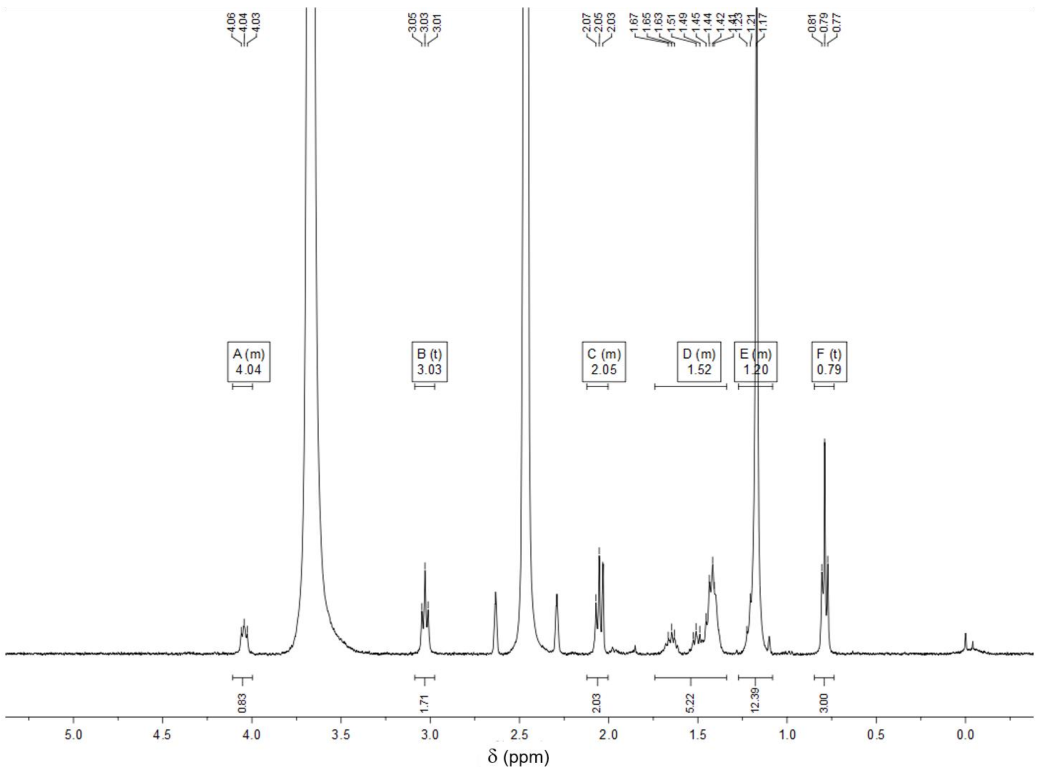

Figure S13 ^{1}H-NMR spectrum of *N*-decanoyl arginine in d_6-DMSO.

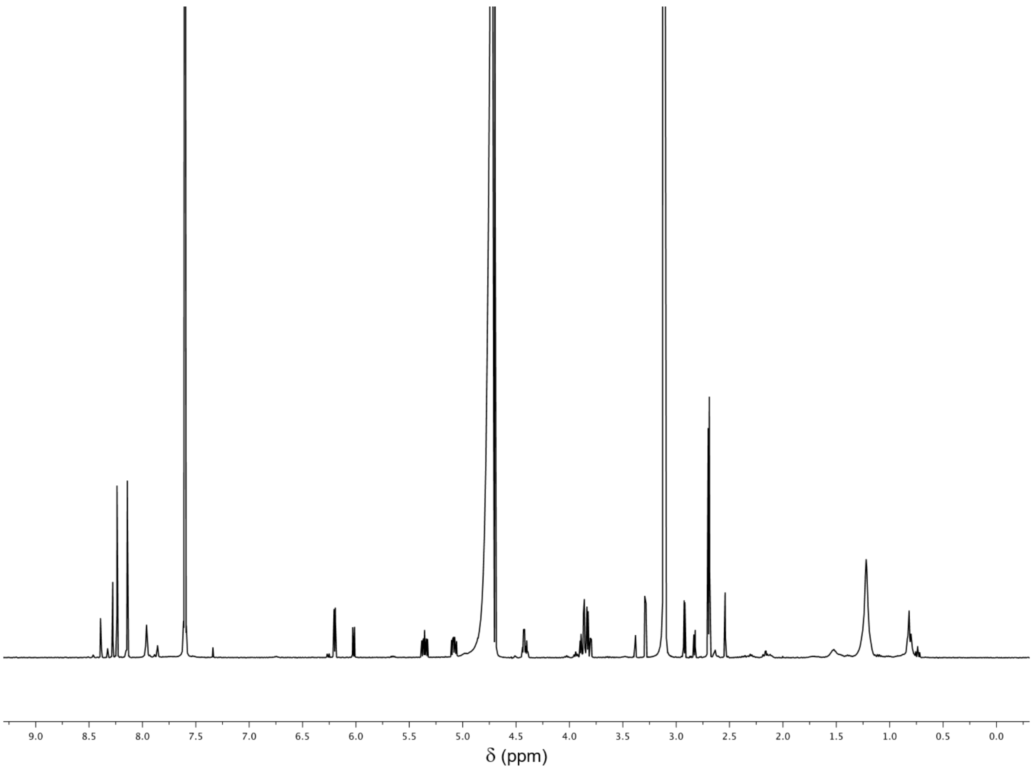

Figure S14 ^{1}H-NMR spectrum showing the activation of 10 mM A3'P after 12 h in the presence of vesicles made of DA:DOH:DHO 4:1:1, 25 mM total lipid concentration, in 100 mM DCI buffer, pH 6.

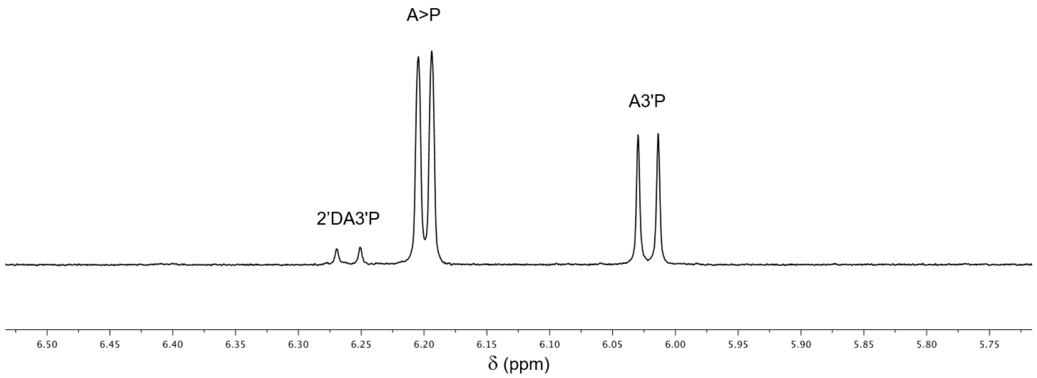

Figure S15 Magnification of the ^{1}H-NMR spectrum showing the activation of 10 mM A3’P after 12 h in the presence of vesicles made of DA:DOH:DHO 4:1:1, 25 mM total lipid concentration, in 100 mM DCI buffer, pH 6. The three signals present in this region correspond to the nucleotide H(1’) resonances of A3’P (right), A>P (middle) and 2’DA3’P (left), respectively.

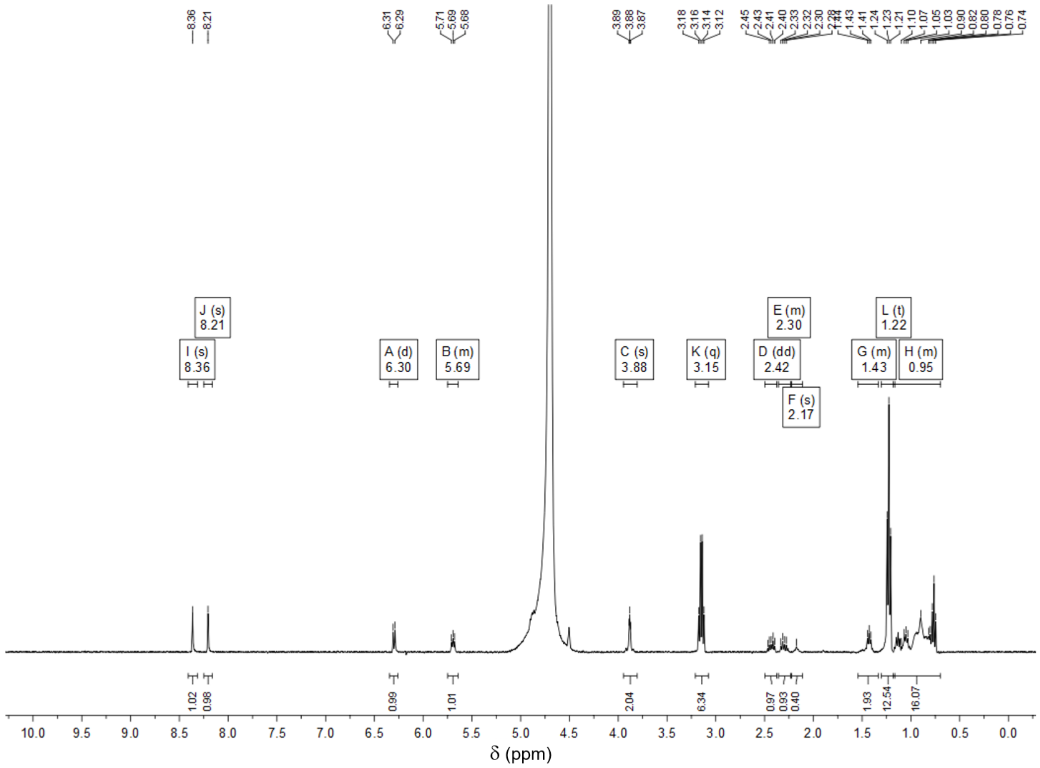

Figure S16 ^{1}H-NMR spectrum of 2′DA3′P in H_2O:D_2O 9:1.

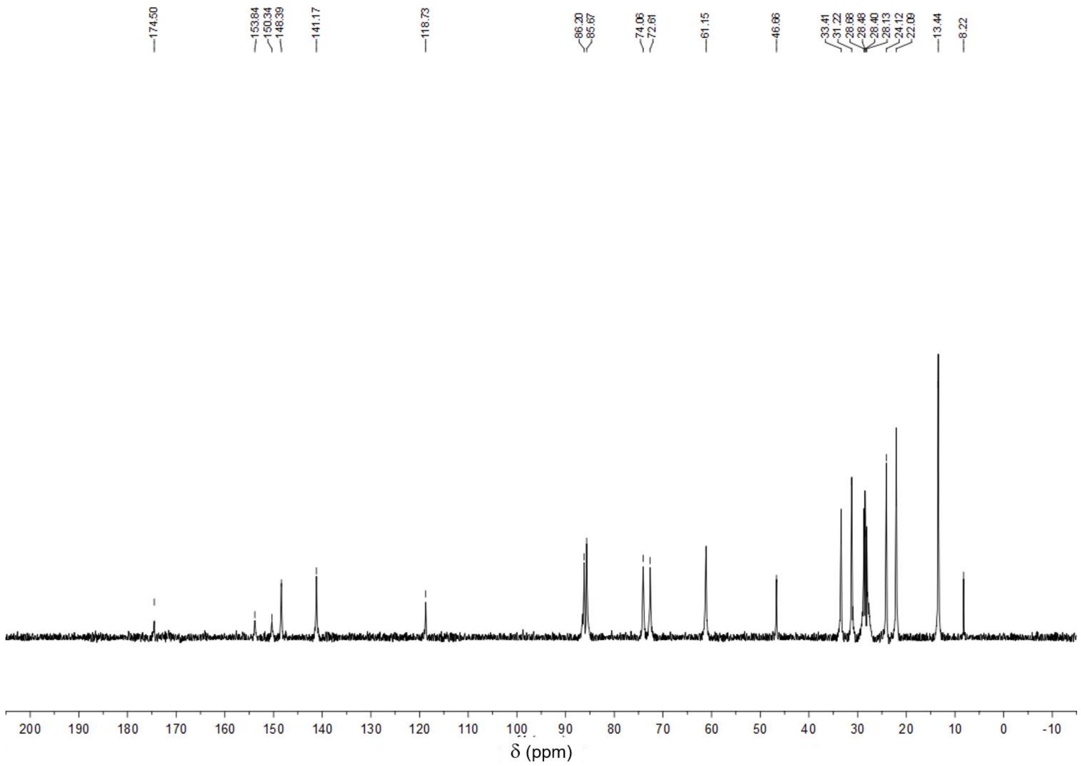

Figure S17 ^{13}C-NMR spectrum of 2′DA3′P in H_2O:D_2O 9:1.

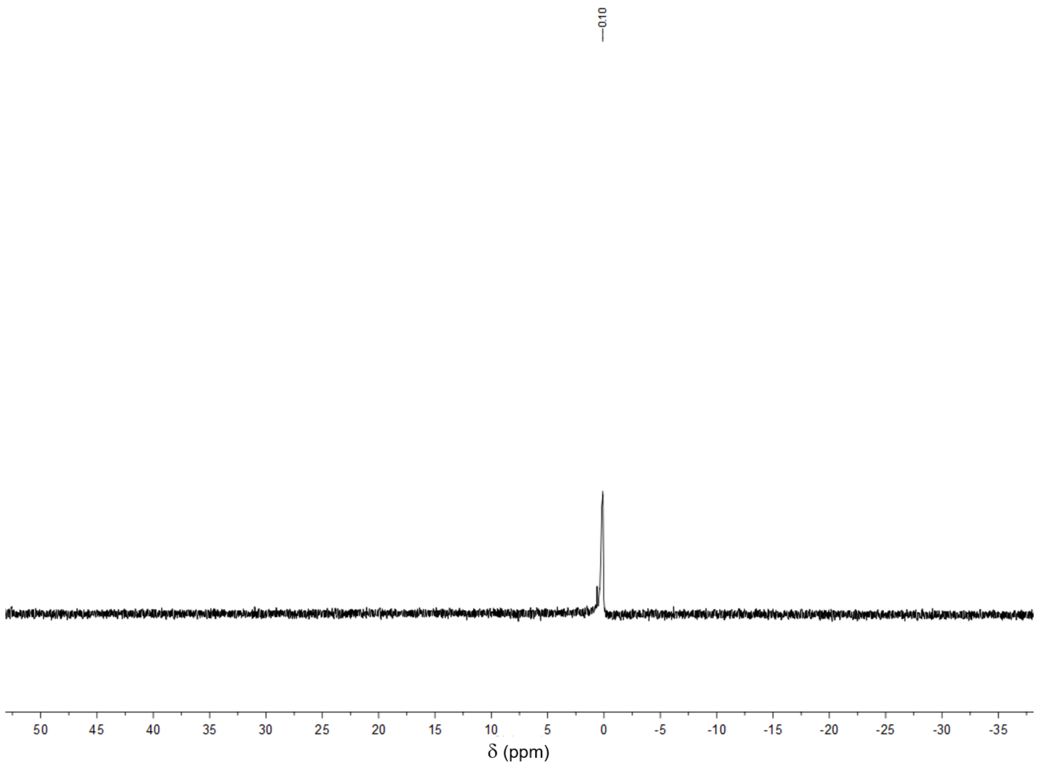

Figure S18 ^{31}P-NMR spectrum of 2′DA3′P in H_2O:D_2O 9:1.

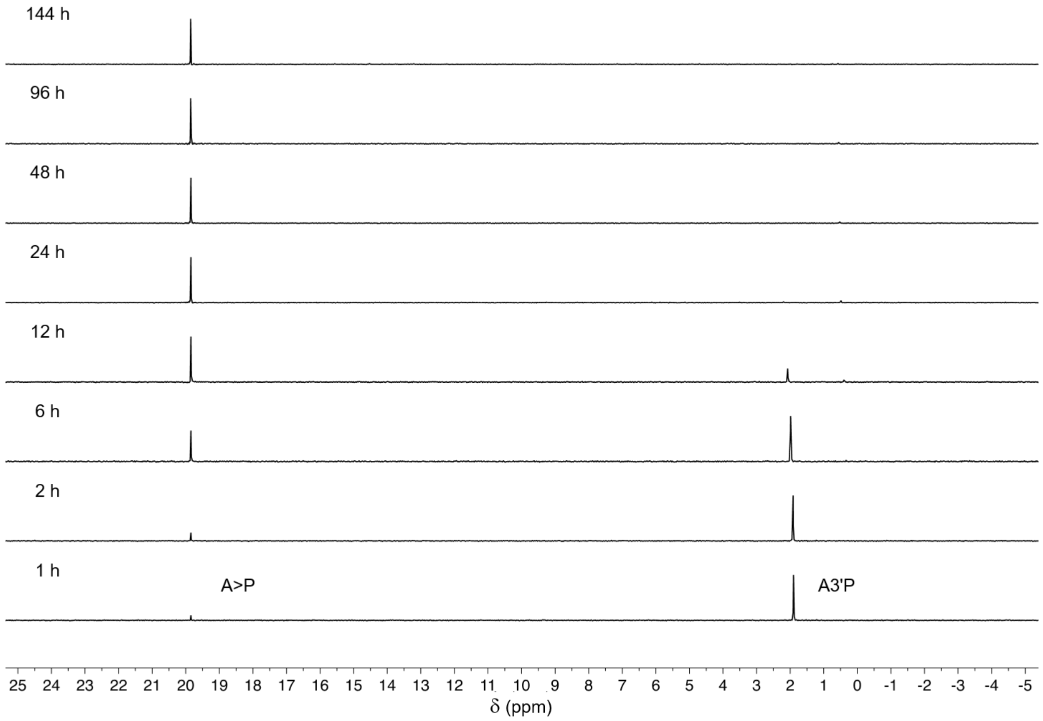

Figure S19 ^{31}P-NMR time course showing the activation of 10 mM A3'P in the presence of vesicles made of DA:DOH:DHO 4:1:1, 25 mM total lipid concentration, in 100 mM DCI buffer, pH 6.

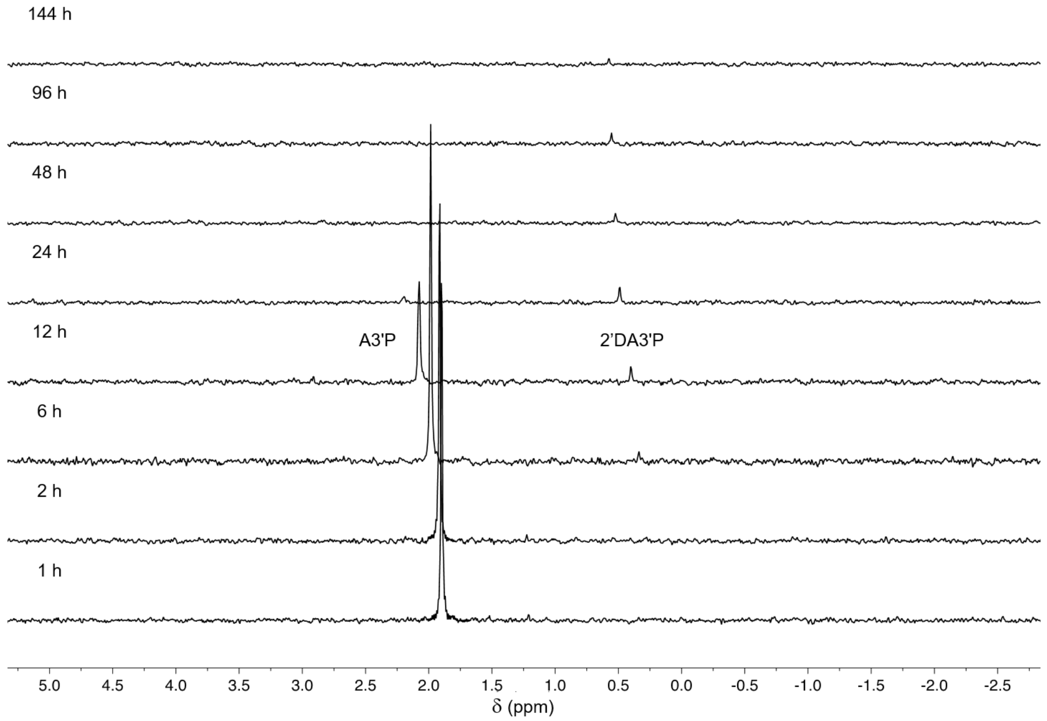

Figure S20 Magnification of the ^{31}P-NMR time course showing the activation of 10 mM A3'P in the presence of vesicles made of DA:DOH:DHO 4:1:1, 25 mM total lipid concentration, in 100 mM DCI buffer, pH 6. The two signals correspond to A3'P (left) and 2'DA3'P (right).

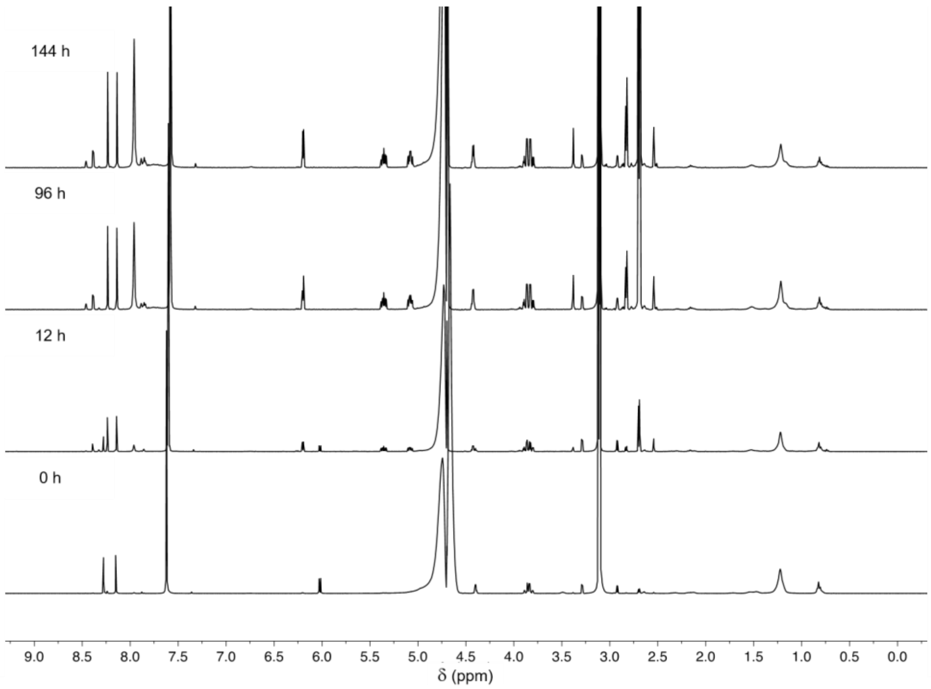

Figure S21 ^{1}H-NMR time course showing the activation of 10 mM A3'P in the presence of vesicles made of DA:DOH:DHO 4:1:1, 25 mM total lipid concentration, in 100 mM DCI buffer, pH 6.

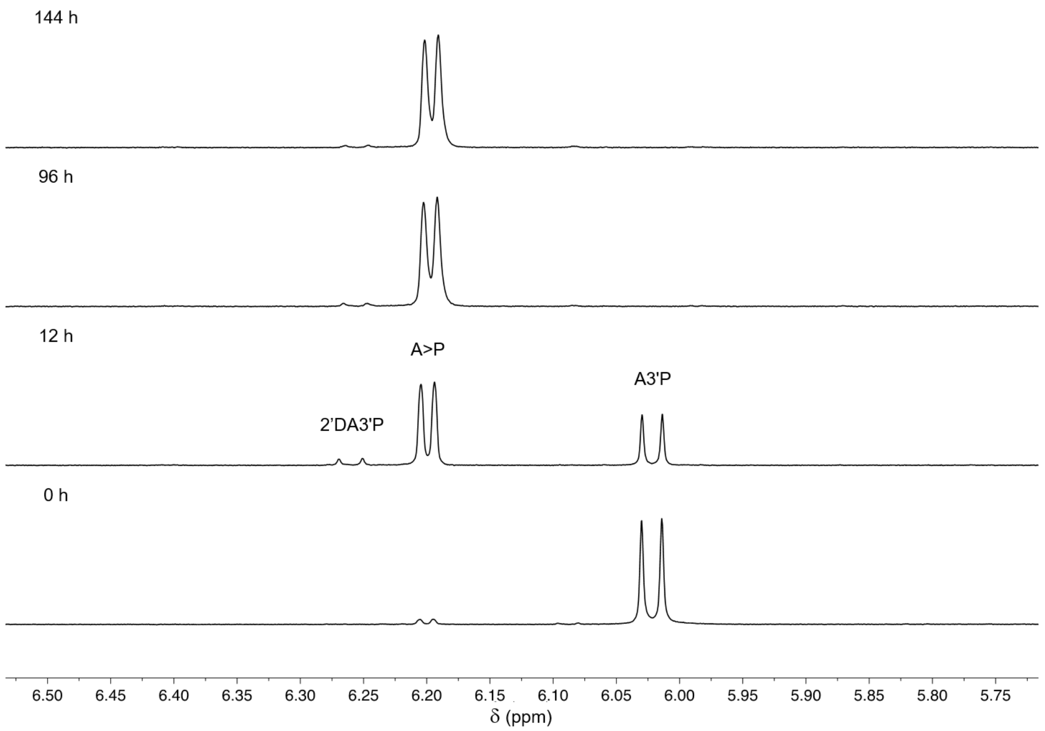

Figure S22 Magnification of the ^{31}P-NMR time course showing the activation of 10 mM A3’P in the presence of vesicles made of DA:DOH:DHO 4:1:1, 25 mM total lipid concentration, in 100 mM DCl buffer, pH 6. The three signals present correspond to the nucleotide H(1’) resonances of A3’P (right), A>P (middle) and 2’DA3’P (left), respectively.

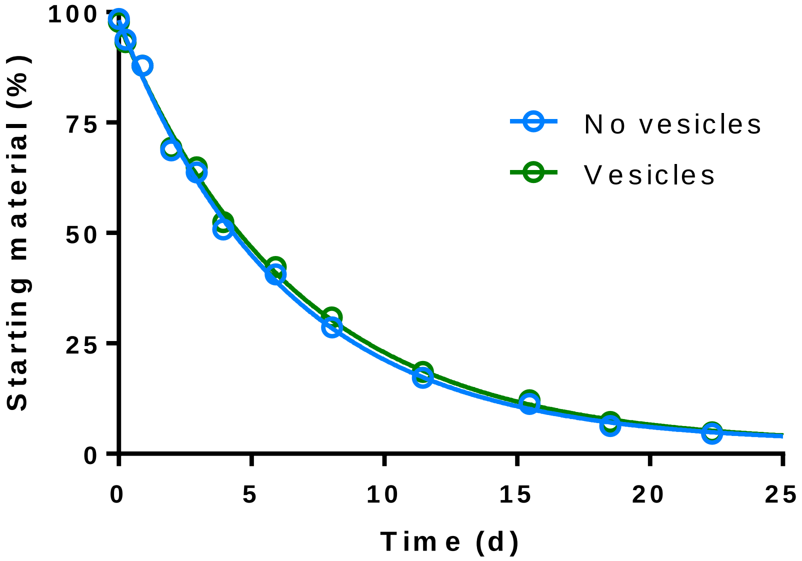

Figure S23 Plot showing the hydrolysis of 2’DA3’P in the presence of vesicles (green line) made of DA:DOH:DHO 4:1:1, 25 mM total lipid concentration, in DCI buffer, pH 6. The hydrolysis of 2’DA3’P in the absence of vesicles (blue line) is shown for comparison.

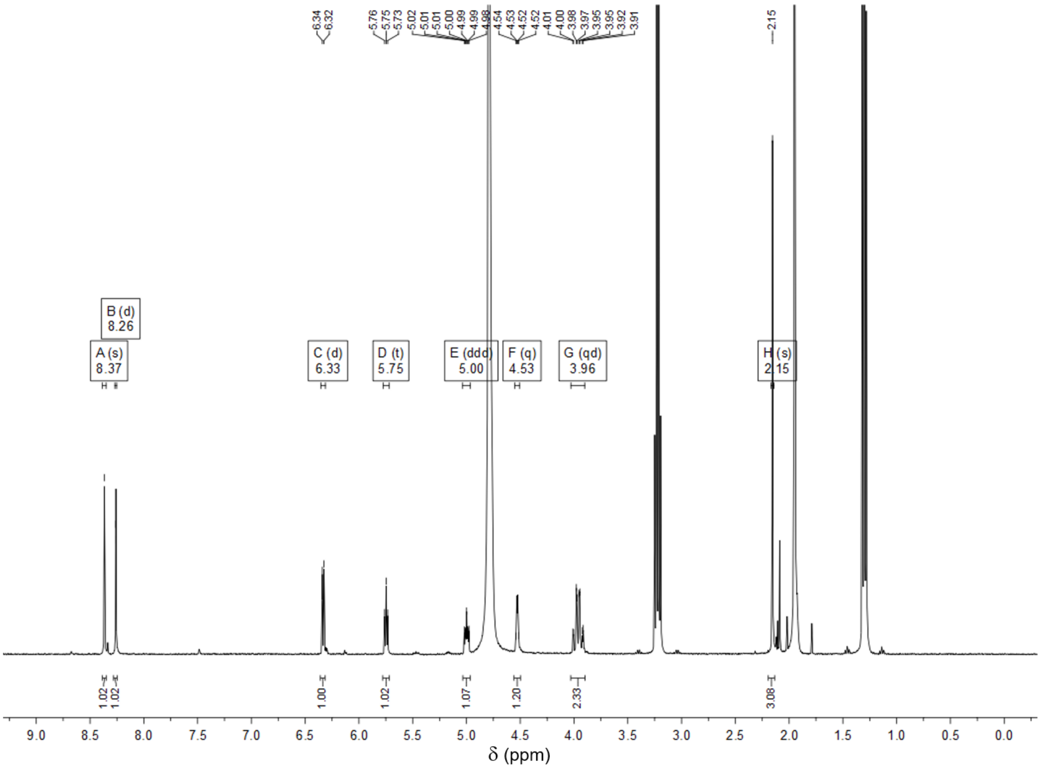

Figure S24 ^{1}H-NMR spectrum of 2′AA3′P in H_2O:D_2O 9:1.

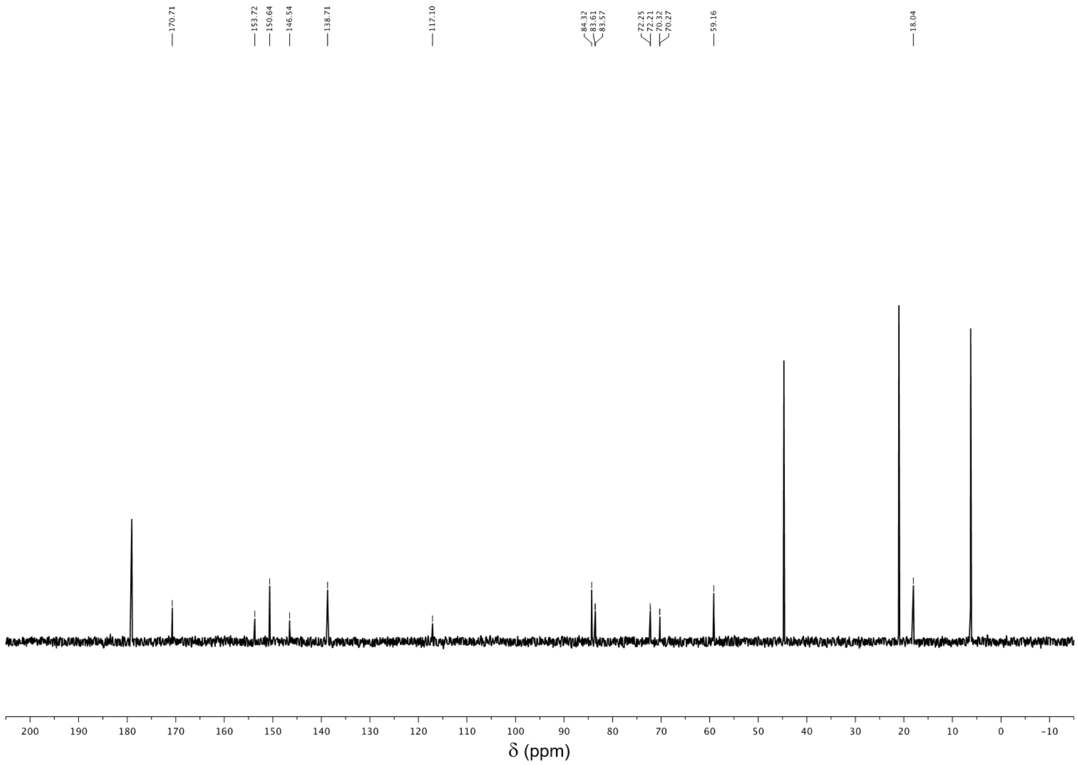

Figure S25 ^{13}C-NMR spectrum of 2’AA3’P in H_2O:D_2O 9:1.

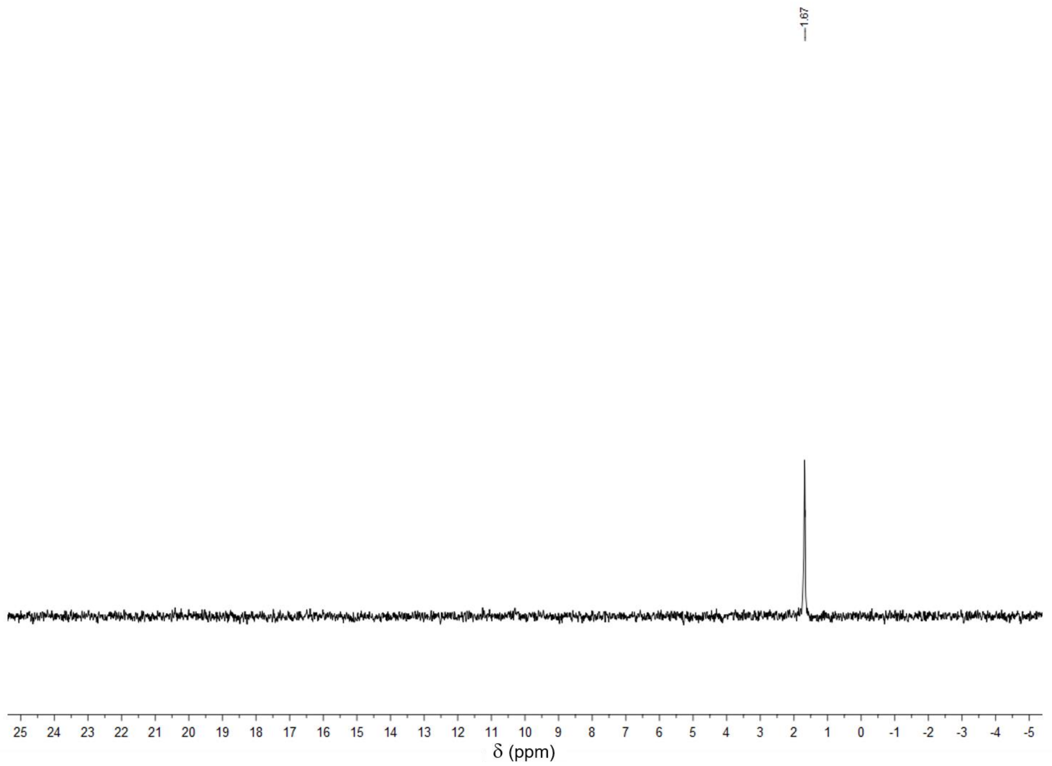

Figure S26 ^{31}P-NMR spectrum of 2′AA3′P in H_2O:D_2O 9:1.

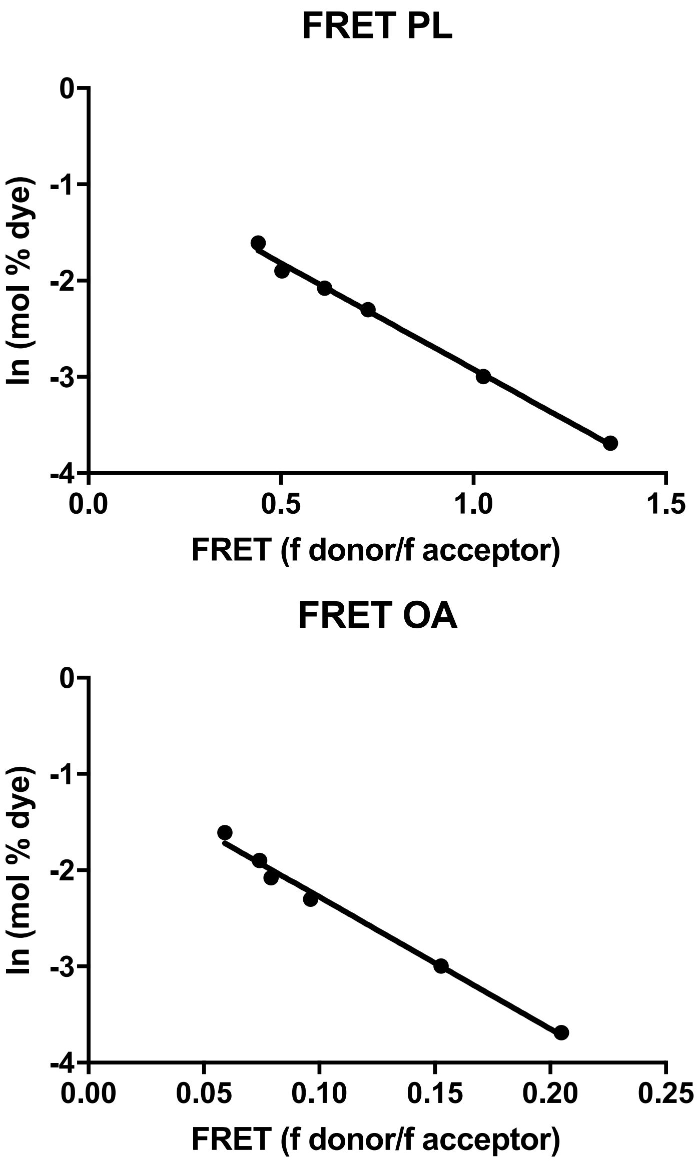

Figure S27 Standard curves of FRET efficiency as a function of mol% FRET dyes in OA (bottom) and POPC (top) vesicles ($R^2 > 0.99$).

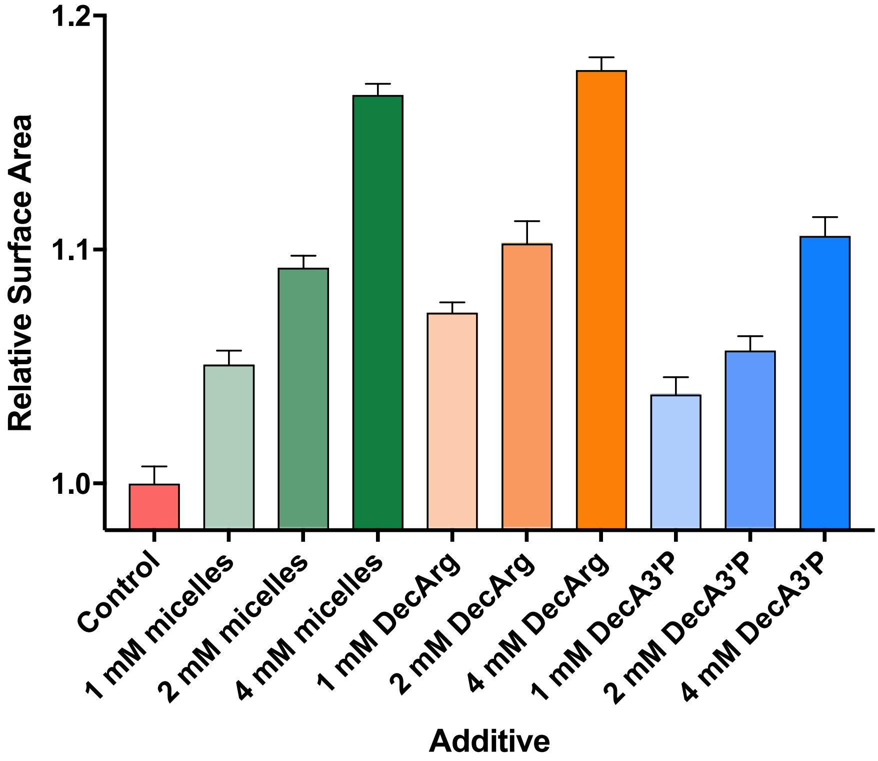

Figure S28 POPC membrane growth upon addition of oleate micelles, *N*-DecArg or 2'DA3'P. Control addition of DCI buffer is also reported. Data are representative of three independent experiments and values are expressed as the mean ± SEM.

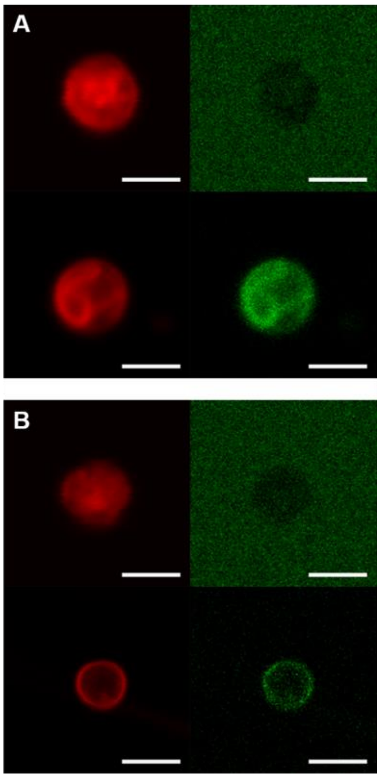

Figure S29 Confocal micrographs of a FAM labeled 7-mer RNA oligonucleotide (green) localised to giant vesicles composed of OA (A) and POPC (B). Lissamine Rhodamine PE dye (red) was used to label vesicle membranes. Top rows show results obtained in the absence of *N*-DecArg (control), bottom rows show results obtained in the presence of *N*-DecArg (25 mol%). Images are contrast adjusted. Scale bars represent 5 µm.

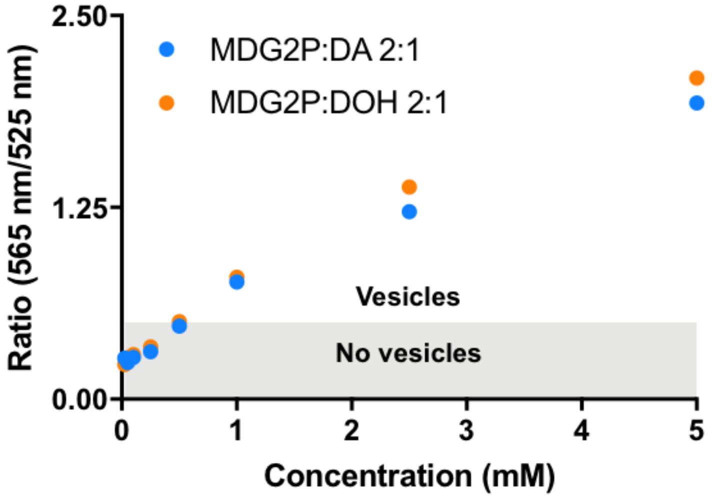

Figure S30 Determination of the critical vesicle concentration of vesicles made of MDG2P:DA 2:1 (blue dots) or MDG2P:DOH 2:1 (orange dots) in 100 mM DCI buffer pH 6, based on the merocyanine 540 assay[7].

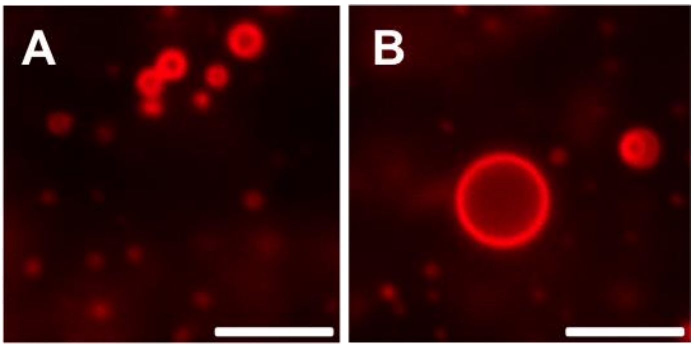

Figure S31 Confocal micrographs of vesicles made of MDG2P:DA 2:1 (A) and MDG2P:DOH 2:1 (B), 15 mM total lipid concentration. Nile red dye was used to label vesicle membranes. The image is contrast adjusted. The scale bar represents 5 µm.

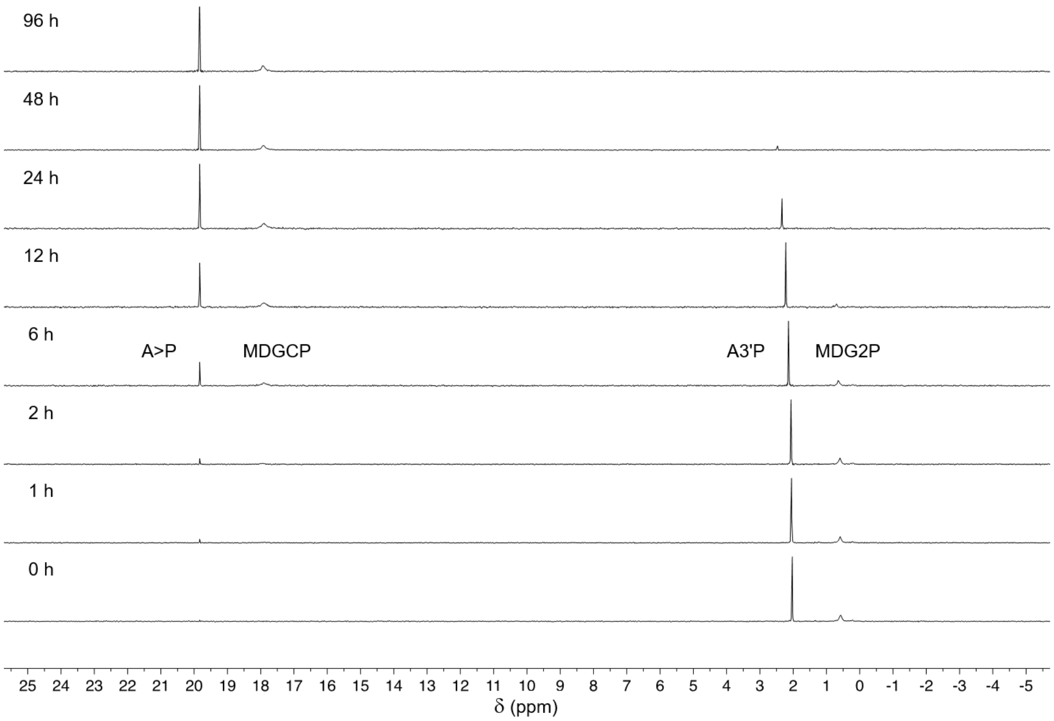

Figure S32 ^{31}P-NMR time course showing the activation of 10 mM A3′P in the presence of vesicles made of MDG2P:DOH 2:1, 15 mM total lipid concentration, in 100 mM DCI buffer, pH 6. A3′P was converted into A>P while MDG2P was converted into MDGCP.

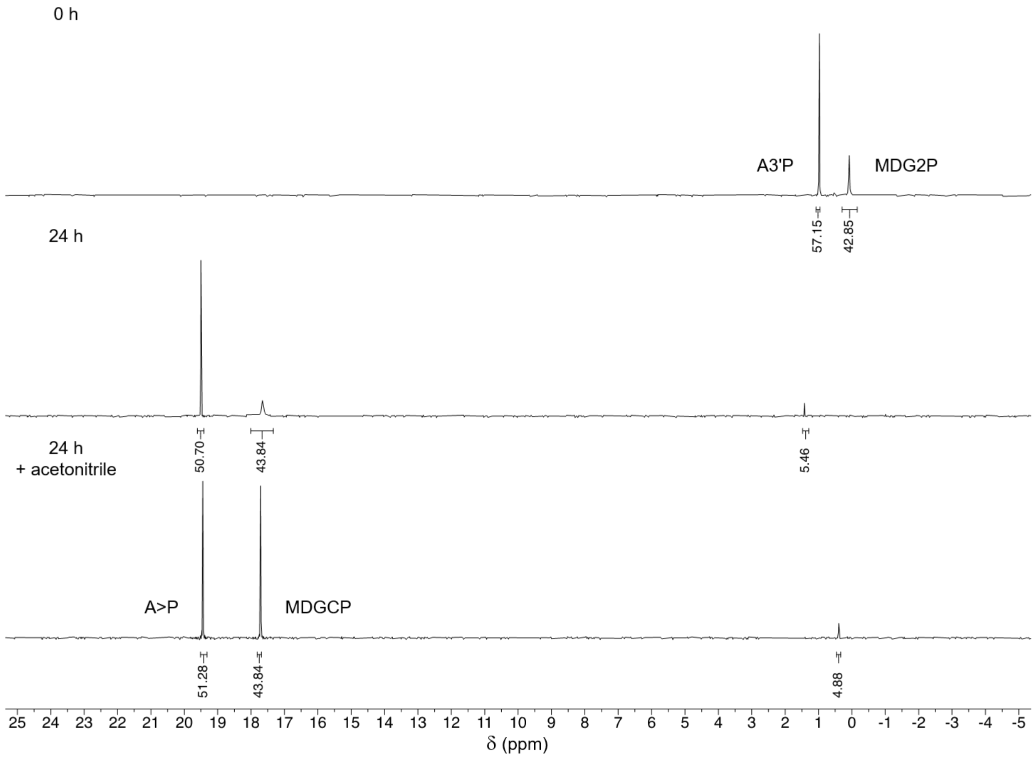

Figure S33 ^{31}P-NMR spectra showing the activation of 10 mM A3'P in the presence of vesicles made of MDG2P:DOH 2:1, 15 mM total lipid concentration, in 100 mM DCI buffer, pH 6. A3'P was converted into A>P while MDG2P was converted into MDGCP. After 24 h, 50% acetonitrile was added to disrupt lipid aggregates.

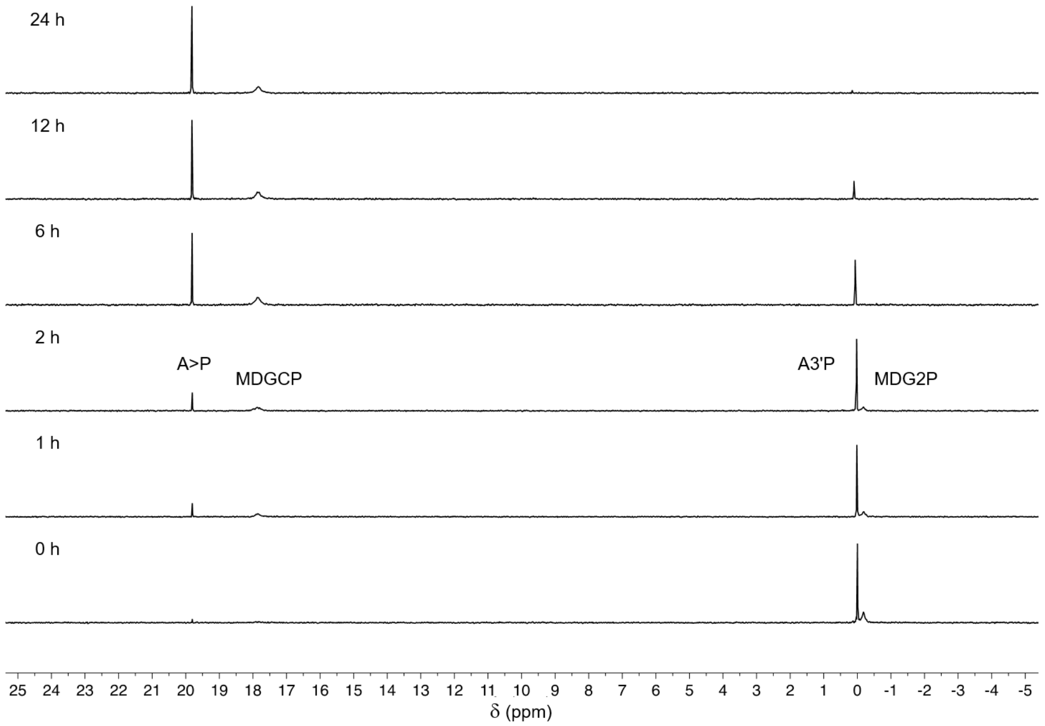

Figure S34 ^{31}P-NMR time course showing the activation of 10 mM A3'P in the presence of vesicles made of MDG2P:DOH 2:1, 15 mM total lipid concentration, in 100 mM DCl buffer, pH 4. A3'P was converted into A>P while MDG2P was converted into MDGCP.

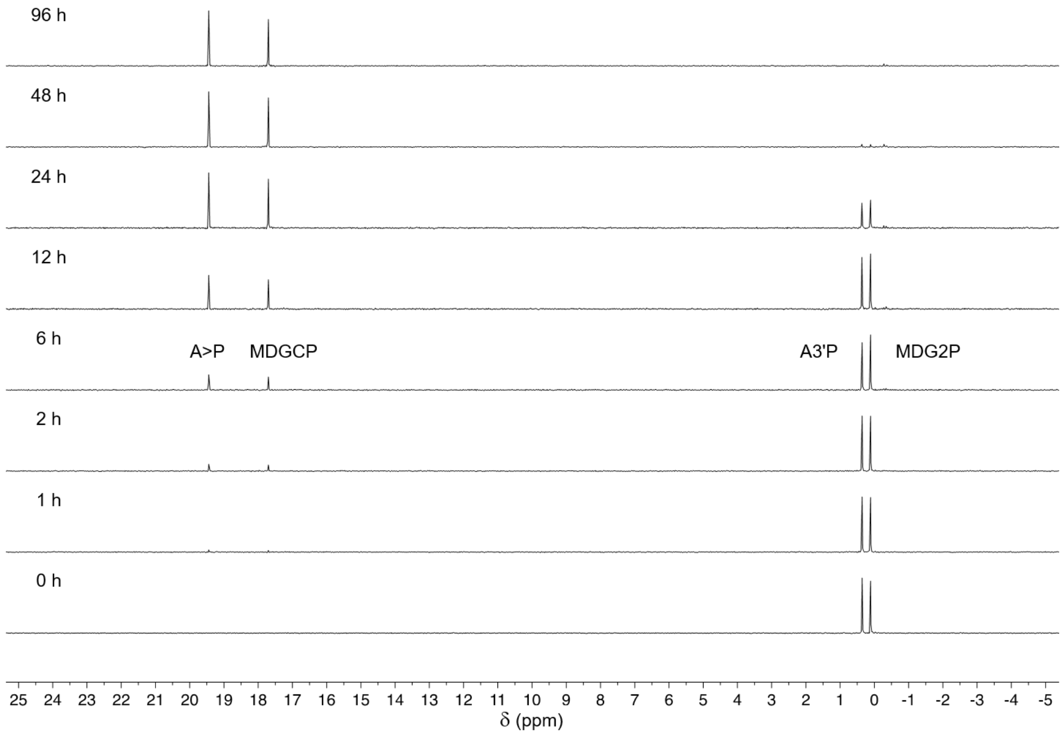

Figure S35 ^{31}P-NMR time course showing the activation of 10 mM A3'P in a 1:1 mixture of 100 mM DCI buffer, pH 6 and acetonitrile. A3'P was converted into A>P while MDG2P was converted into MDGCP.

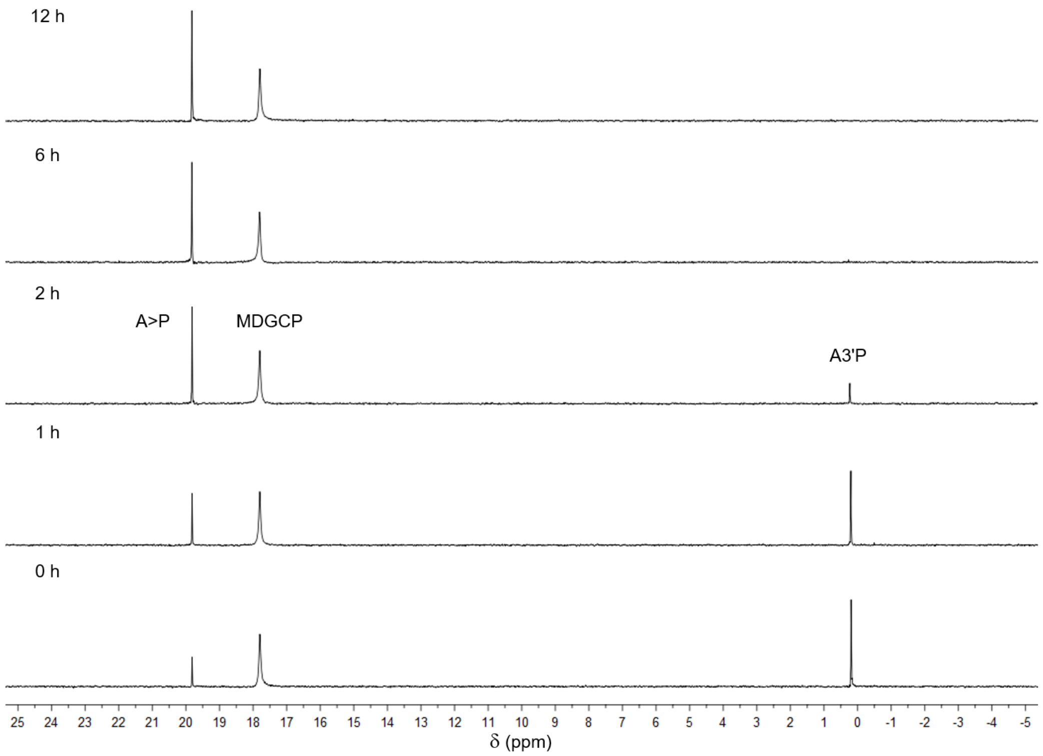

Figure S36 ^{31}P-NMR time course showing the activation of 10 mM A3'P in the presence of vesicles made of MDGCP:DOH 2:1, 15 mM total lipid concentration, in 100 mM DCl buffer, pH 4.

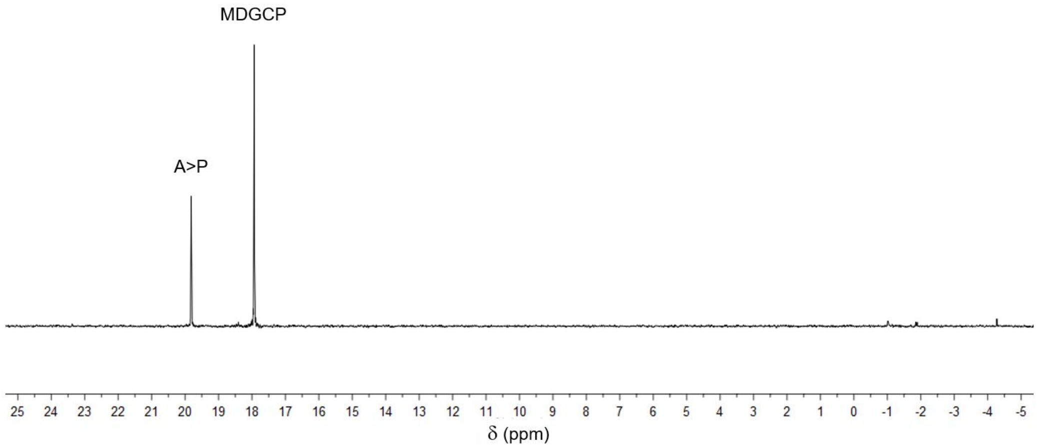

Figure S37 ^{31}P-NMR spectrum showing the activation of 10 mM A3'P after 0.5 h driven by methyl isocyanide and acetaldehyde in the presence of vesicles made of MDGCP:DOH 2:1, 15 mM total lipid concentration, in 100 mM imidazole buffer, pH 6.5. A3'P was quantitatively converted into A>P.

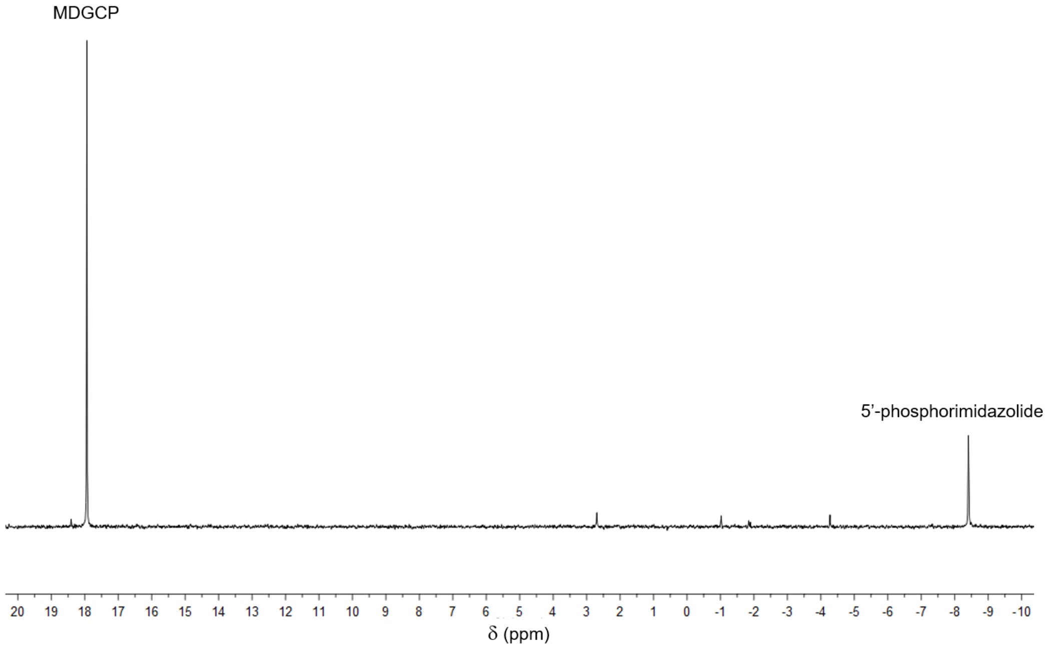

Figure S38 ^{31}P-NMR spectrum showing the activation of 10 mM A5’P after 0.5 h driven by methyl isocyanide and acetaldehyde in the presence of vesicles made of MDGCP:DOH 2:1, 15 mM total lipid concentration, in 100 mM imidazole buffer, pH 6.5. A5’P was largely converted (93%) into the corresponding A5’-phosphorimidazolide.

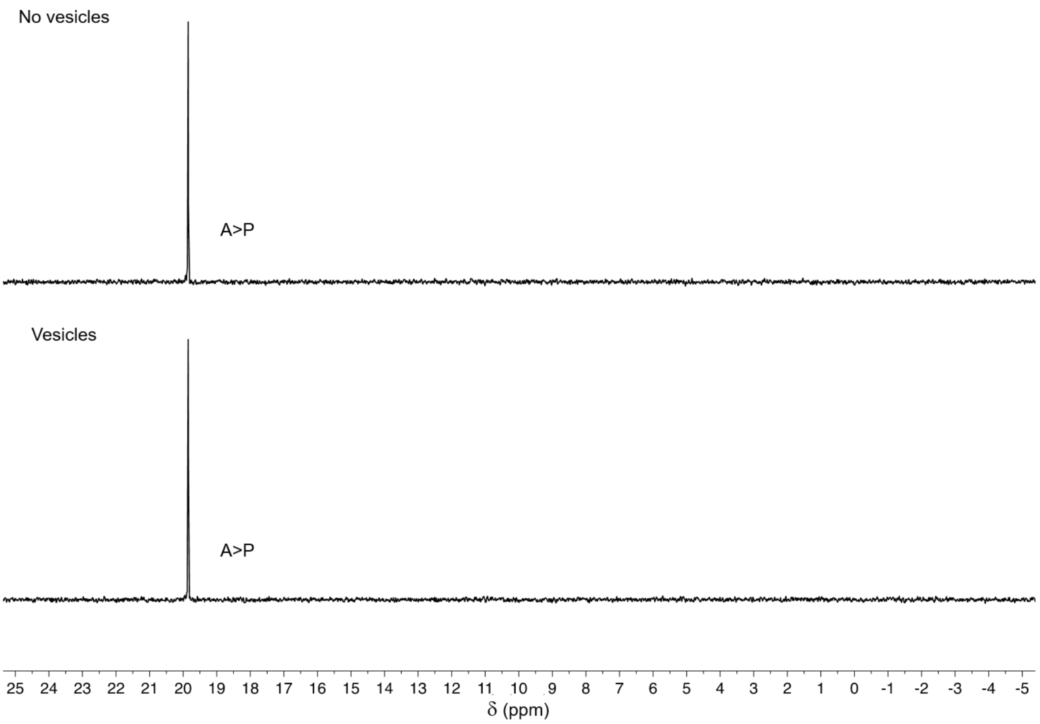

Figure S39 ^{31}P-NMR spectrum showing the activation of 10 mM A3'P after 96 h in the presence of 100 mM AcGly and vesicles made of DA:DOH:DHO 4:1:1, 25 mM total lipid concentration, in 100 mM DCI buffer, pH 6 (bottom). The ^{31}P-NMR spectrum showing the activation of 10 mM A3'P after 96 h in the presence of 100 mM AcGly without vesicles (top) is provided for comparison.

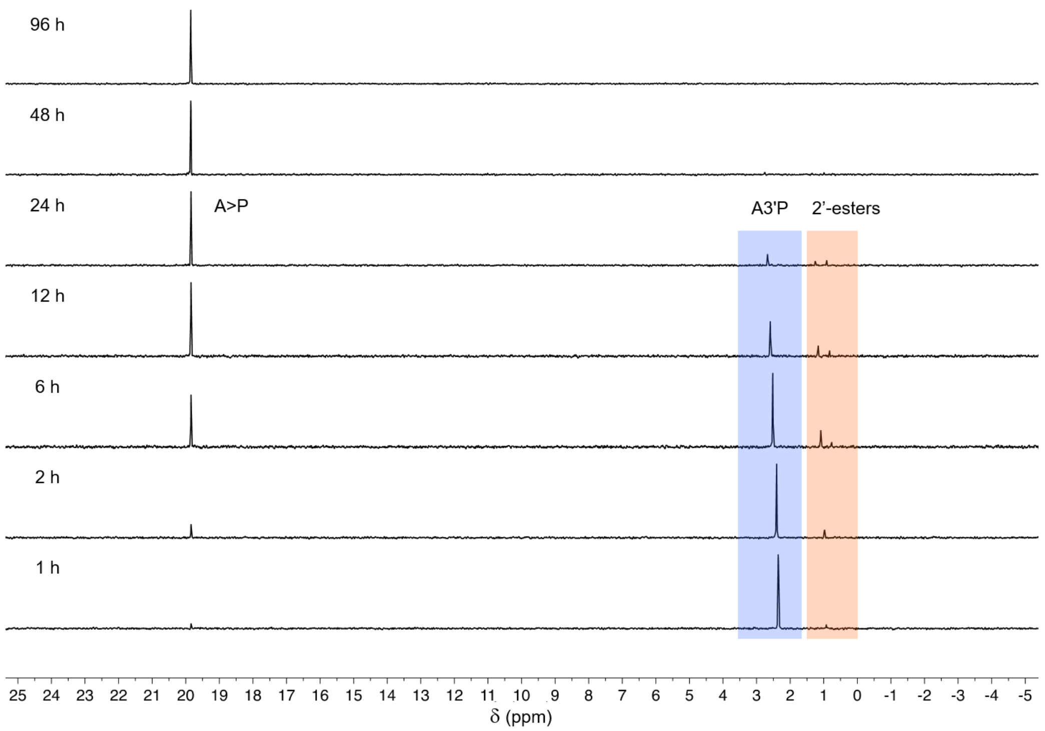

Figure S40 ^{31}P-NMR time course showing the activation of 10 mM A3'P in the presence of 100 mM AcGly and vesicles made of DA:DOH:DHO 4:1:1, 25 mM total lipid concentration, in 100 mM DCl buffer, pH 6.

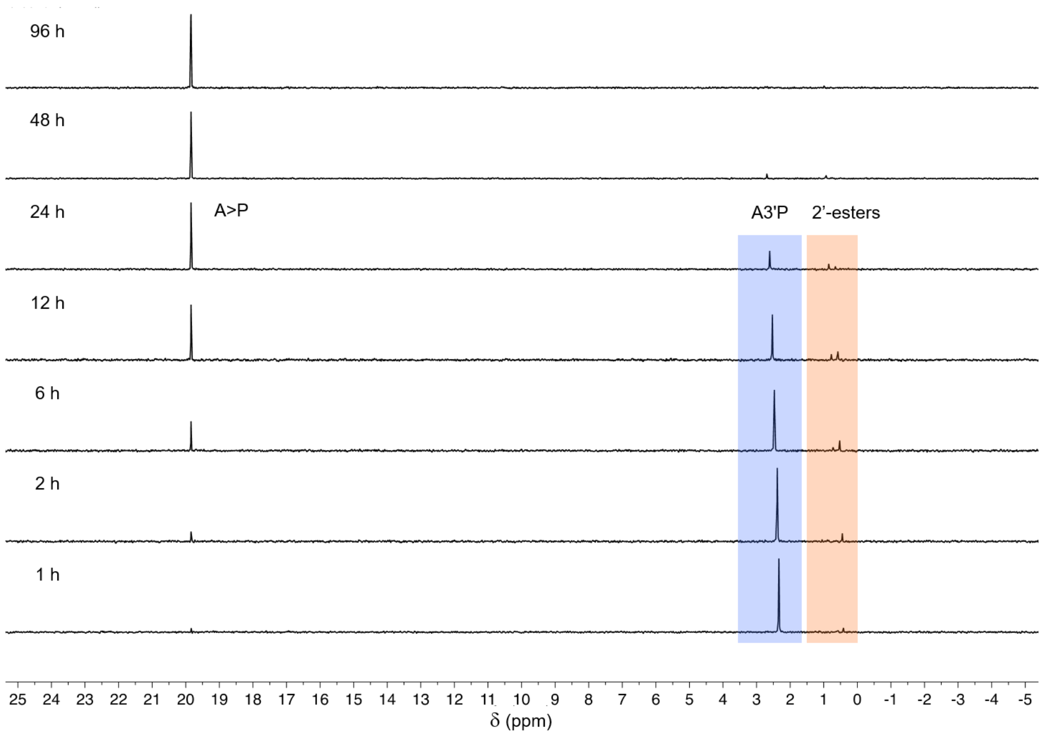

Figure S41 ^{31}P-NMR time course showing the activation of 10 mM A3'P in the presence of 100 mM AcAla and vesicles made of DA:DOH:DHO 4:1:1, 25 mM total lipid concentration, in 100 mM DCl buffer, pH 6.

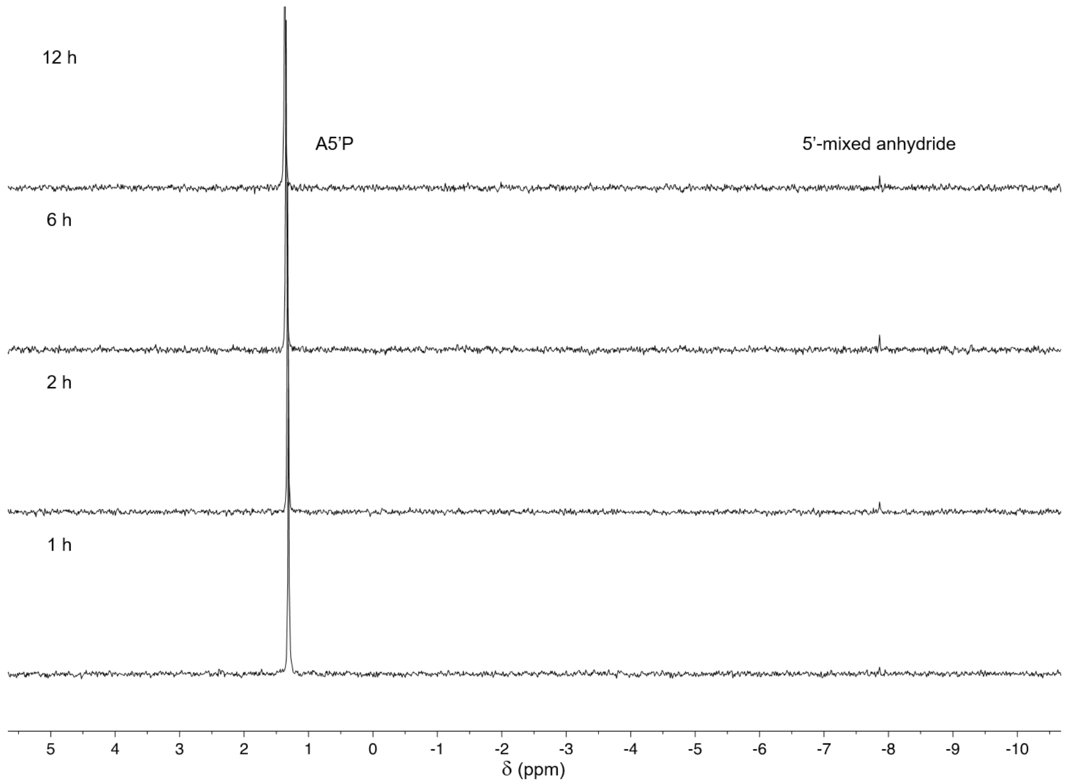

Figure S42 ^{31}P-NMR time course showing the activation of 10 mM A5’P in the presence of 100 mM AcGly and vesicles made of DA:DOH:DHO 4:1:1, 25 mM total lipid concentration, in 100 mM DCl buffer, pH 6.

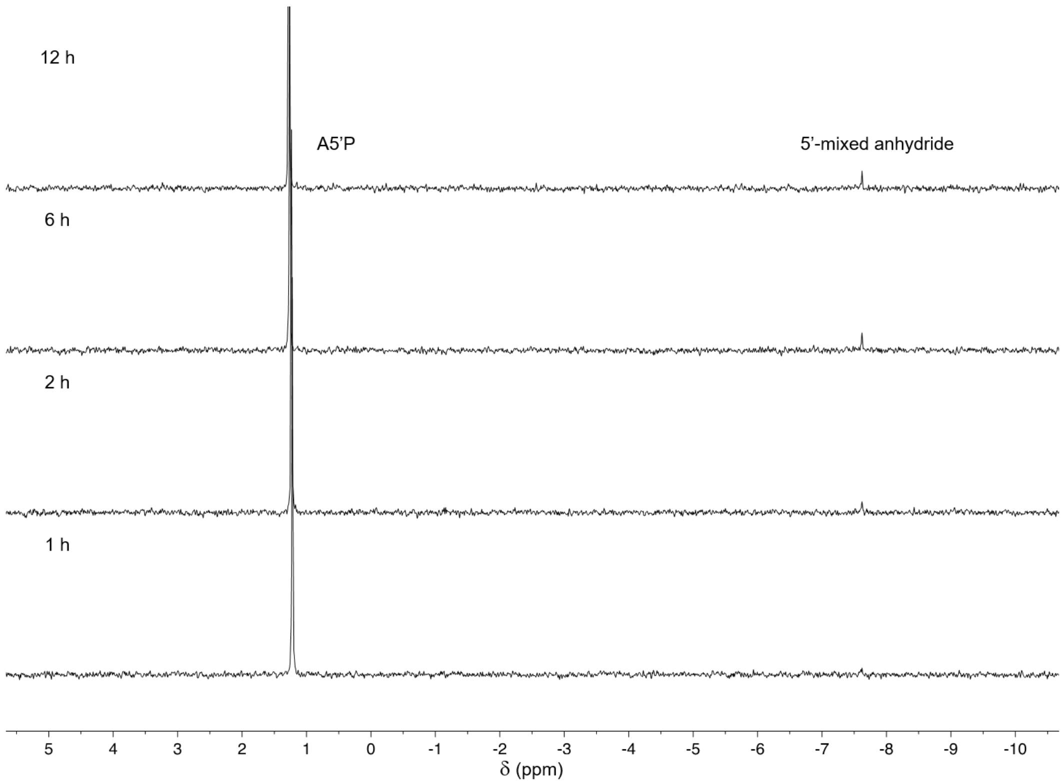

Figure S43 ^{31}P-NMR time course showing the activation of 10 mM A5'P in the presence of 100 mM AcAla and vesicles made of DA:DOH:DHO 4:1:1, 25 mM total lipid concentration, in 100 mM DCI buffer, pH 6.

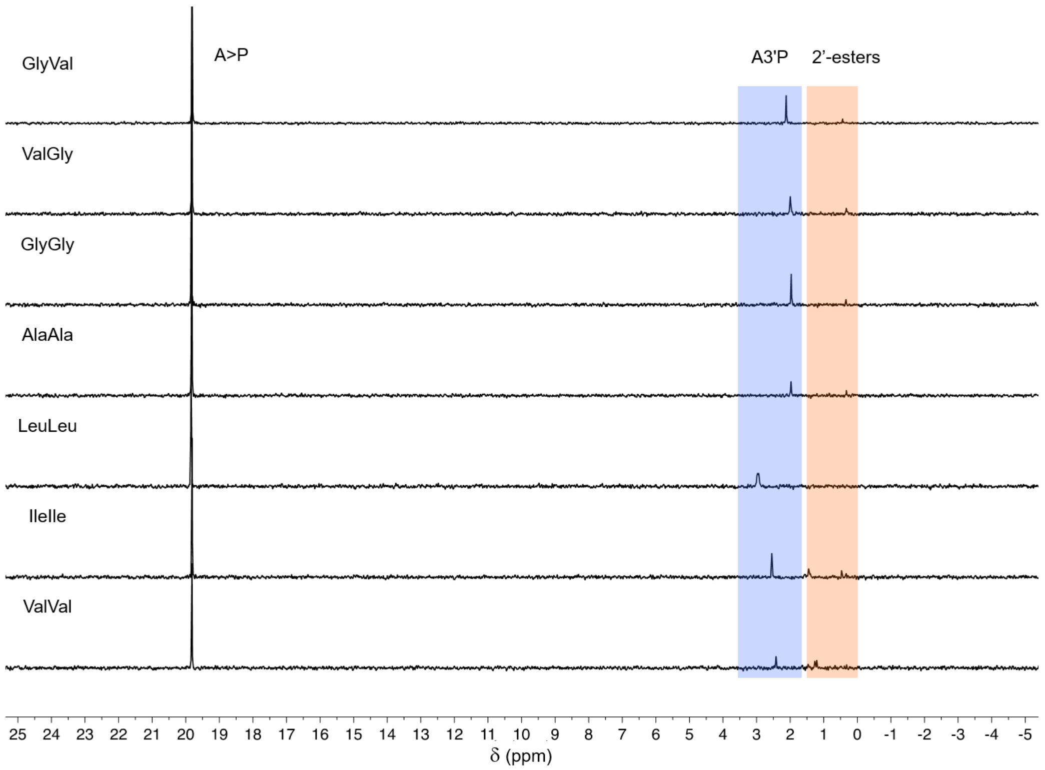

Figure S44 ^{31}P-NMR spectra showing the products obtained upon activation of 10 mM A3'P after 24 h in the presence of 100 mM dipeptides and vesicles made of DA:DOH:DHO 4:1:1, 25 mM total lipid concentration, in 100 mM DCl buffer, pH 6. Selected dipeptides: GlyVal, ValGly, GlyGly, AlaAla, LeuLeu, IleIle, ValVal.

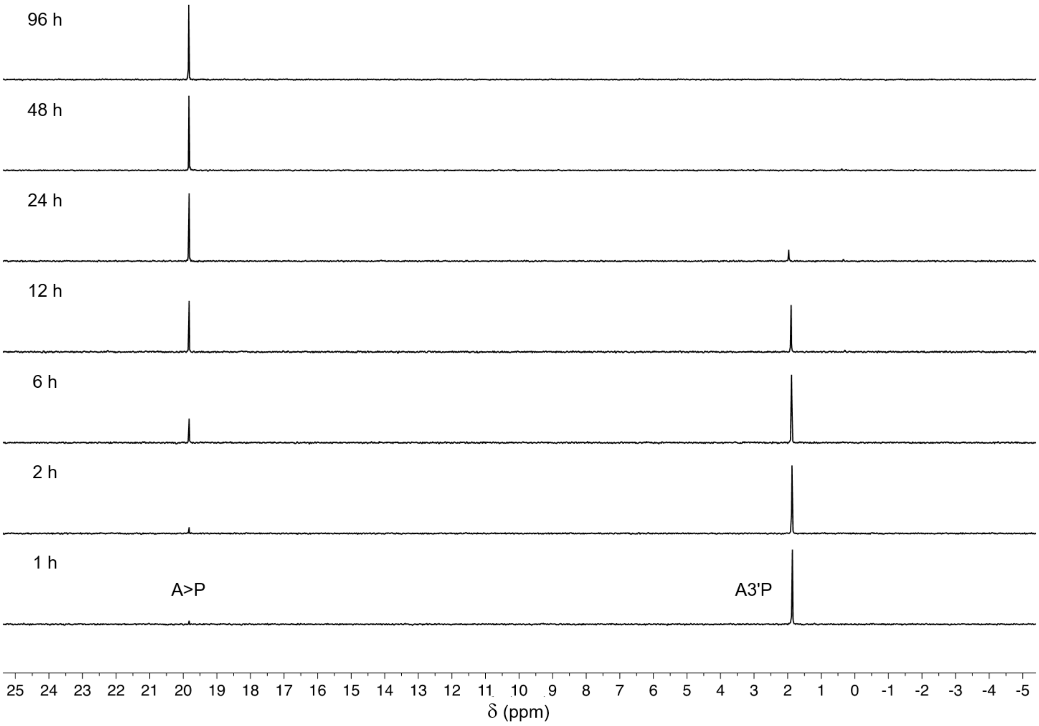

Figure S45 ^{31}P-NMR time course showing the activation of 10 mM A3’P in the presence of 100 mM GlyGly and vesicles made of DA:DOH:DHO 4:1:1, 25 mM total lipid concentration, in 100 mM DCl buffer, pH 6.

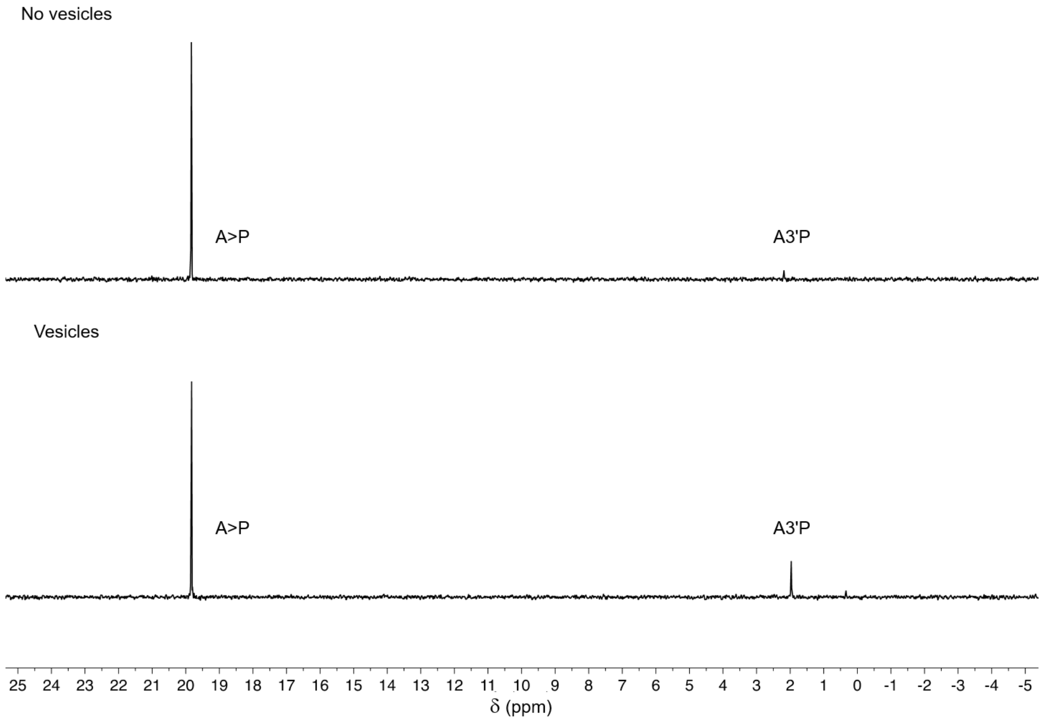

Figure S46 ^{31}P-NMR spectrum showing the activation of 10 mM A3’P after 24 h in the presence of 100 mM GlyGly and vesicles made of DA:DOH:DHO 4:1:1, 25 mM total lipid concentration, in 100 mM DCI buffer, pH 6 (bottom). The ^{31}P-NMR spectrum showing the activation of 10 mM A3’P after 24 h in the presence of 100 mM GlyGly without vesicles (top) is provided for comparison.

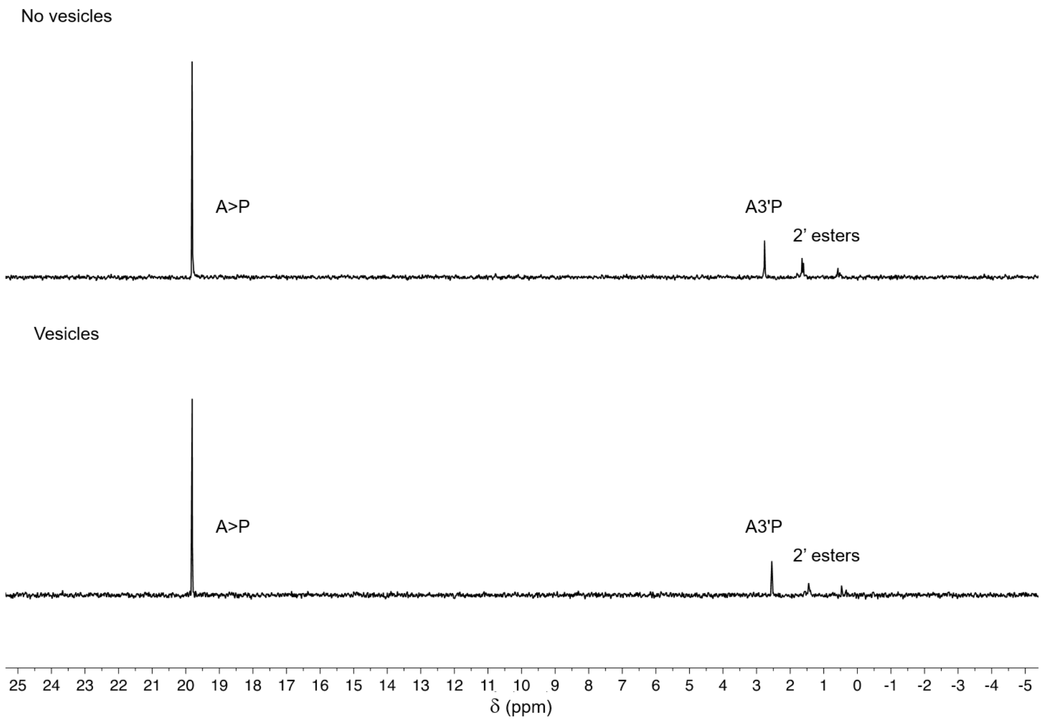

Figure S47 ^{31}P-NMR spectrum showing the activation of 10 mM A3'P after 24 h in the presence of 100 mM IleIle and vesicles made of DA:DOH:DHO 4:1:1, 25 mM total lipid concentration, in 100 mM DCI buffer, pH 6 (bottom). The ^{31}P-NMR spectrum showing the activation of 10 mM A3'P after 24 h in the presence of 100 mM IleIle without vesicles (top) is provided for comparison.

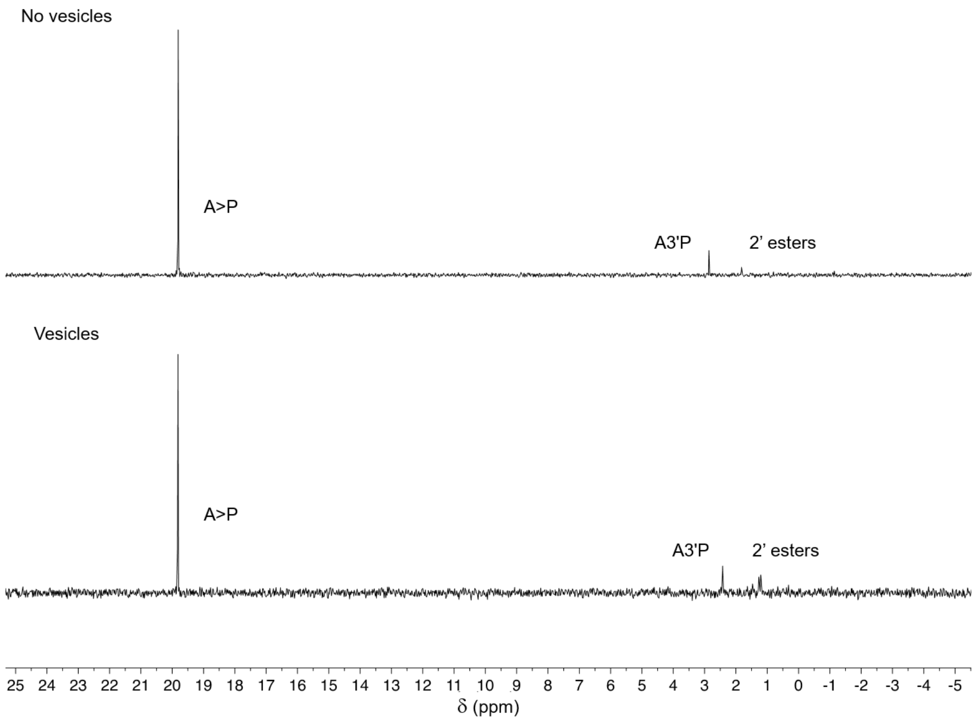

Figure S48 ^{31}P-NMR spectrum showing the activation of 10 mM A3'P after 24 h in the presence of 100 mM ValVal and vesicles made of DA:DOH:DHO 4:1:1, 25 mM total lipid concentration, in 100 mM DCI buffer, pH 6 (bottom). The ^{31}P-NMR spectrum showing the activation of 10 mM A3'P after 24 h in the presence of 100 mM ValVal without vesicles (top) is provided for comparison.

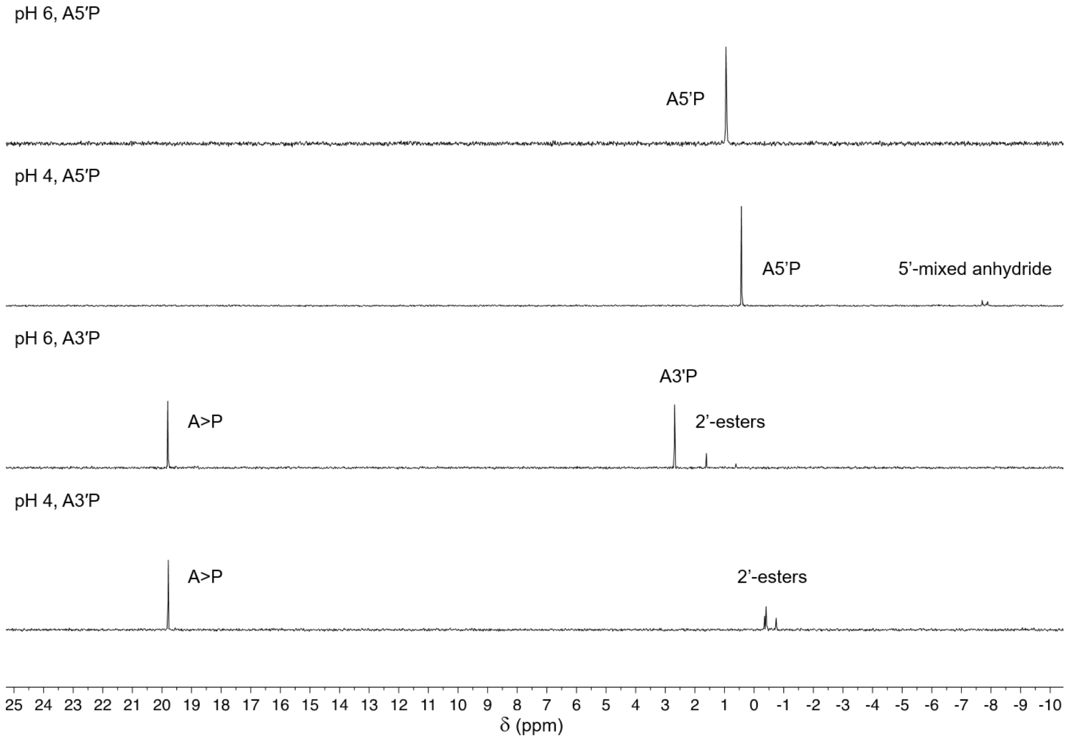

Figure S49 ^{31}P-NMR spectrum showing the activation of 10 mM A3′P (bottom) or A5′P (top) after 12 h in the presence of 100 mM ValVal in 100 mM DCI buffer, pH 4 and 6.

Supplementary references

1. Rasband, W.S. ImageJ. http://rsb.info.nih.gov/ij/.
2. Gibard, C., Bhowmik, S., Karki, M., Kim, E.-K., and Krishnamurthy, R. (2018). Phosphorylation, oligomerization and self-assembly in water under potential prebiotic conditions. Nat. Chem. *10*, 212–217.
3. Joshi, A., Shaikh, M., Singh, S., Rajendran, A., Mhetre, A., and Kamat, S.S. (2018). Biochemical characterization of the PHARC-associated serine hydrolase ABHD12 reveals its preference for very-long-chain lipids. J. Biol. Chem. *293*, 16953–16963.
4. Liu, B., Cui, C., Duan, W., Zhao, M., Peng, S., Wang, L., Liu, H., and Cui, G. (2009). Synthesis and evaluation of anti-tumor activities of N 4 fatty acyl amino acid derivatives of 1-β-arabinofuranosylcytosine. Eur. J. Med. Chem. *44*, 3596–3600.
5. Hanczyc, M.M., Fujikawa, S.M., and Szostak, J.W. (2003). Experimental models of primitive cellular compartments: encapsulation, growth, and division. Science *302*, 618–22.
6. Zhu, T.F., Budin, I., and Szostak, J.W. (2013). Preparation of fatty acid or phospholipid vesicles by thin-film rehydration. Methods Enzymol. *533*, 267–274.
7. Williamson, P., Mattocks, K., and Schlegel, R.A. (1983). Merocyanine 540, a fluorescent probe sensitive to lipid packing. BBA - Biomembr. *732*, 387–393.
8. Paprocki, D., Koszelewski, D., Walde, P., and Ostaszewski, R. (2015). Efficient Passerini reactions in an aqueous vesicle system. RSC Adv. *5*, 102828–102835.
